# Effects of Climate Variability and Accelerated Forest Thinning on Watershed-Scale Runoff in Southwestern USA Ponderosa Pine Forests

**DOI:** 10.1371/journal.pone.0111092

**Published:** 2014-10-22

**Authors:** Marcos D. Robles, Robert M. Marshall, Frances O'Donnell, Edward B. Smith, Jeanmarie A. Haney, David F. Gori

**Affiliations:** 1 The Nature Conservancy Center for Science and Public Policy, Tucson, Arizona, United States of America; 2 Northern Arizona University, Flagstaff, Arizona, United States of America; 3 The Nature Conservancy in California, Sacramento, California, United States of America; 4 The Nature Conservancy in New Mexico, Santa Fe, New Mexico, United States of America; Oregon State University, United States of America

## Abstract

The recent mortality of up to 20% of forests and woodlands in the southwestern United States, along with declining stream flows and projected future water shortages, heightens the need to understand how management practices can enhance forest resilience and functioning under unprecedented scales of drought and wildfire. To address this challenge, a combination of mechanical thinning and fire treatments are planned for 238,000 hectares (588,000 acres) of ponderosa pine (*Pinus ponderosa*) forests across central Arizona, USA. Mechanical thinning can increase runoff at fine scales, as well as reduce fire risk and tree water stress during drought, but the effects of this practice have not been studied at scales commensurate with recent forest disturbances or under a highly variable climate. Modifying a historical runoff model, we constructed scenarios to estimate increases in runoff from thinning ponderosa pine at the landscape and watershed scales based on driving variables: pace, extent and intensity of forest treatments and variability in winter precipitation. We found that runoff on thinned forests was about 20% greater than unthinned forests, regardless of whether treatments occurred in a drought or pluvial period. The magnitude of this increase is similar to observed declines in snowpack for the region, suggesting that accelerated thinning may lessen runoff losses due to warming effects. Gains in runoff were temporary (six years after treatment) and modest when compared to mean annual runoff from the study watersheds (0–3%). Nonetheless gains observed during drought periods could play a role in augmenting river flows on a seasonal basis, improving conditions for water-dependent natural resources, as well as benefit water supplies for downstream communities. Results of this study and others suggest that accelerated forest thinning at large scales could improve the water balance and resilience of forests and sustain the ecosystem services they provide.

## Introduction

As we transition into a warmer century, managing forests in the southwestern United States for resilience is more urgent and necessary than ever. Forest conditions across the region have declined significantly. In ponderosa pine (*Pinus ponderosa*) forests of central Arizona, stand densities range from 2 to 44 times greater than during pre-settlement conditions and total basal areas range from 2 to 4 times greater [1]–[3]. Severe wildfires and drought have caused high tree mortality rates across 14–18% of forests and woodlands in Arizona and New Mexico [4] and these results do not include mortality from two high-severity wildfires in 2011, the Wallow and the Las Conchas wildfires, which burned 217,000 and 63,500 ha (535,000 and 157,000 acres) in Arizona and New Mexico, respectively. Persistent drought conditions have also adversely impacted water-dependent habitats and species within and downstream of forest uplands, as evidenced by heightened mortality of a riparian tree species in central Arizona [5] and declines in number of species and densities of native fish in the Gila River in southwestern New Mexico [6], [7]. Researchers suggest that warming is amplifying the size and severity of wildfire- and drought-mortality events and decreasing snow pack levels [8]–[11]. Suggested mechanisms involve changes in the water cycle: increased evapotranspiration losses, extended water-stress periods, earlier snowmelt, and lengthened fire seasons. These changes and trends suggest a widespread drying of forests and an increasing risk of uncharacteristic fire and competition-induced water stress and mortality.

At the same time, many communities in the western U.S. are facing critical water shortages and river flows in some basins have diminished or been lost altogether due to unsustainable water management practices [12]–[15]. Recent studies at the scale of both the Colorado River basin and the state of Arizona concluded that water supplies for many communities will be inadequate to meet future projected demands [16],[17]. Moreover, elevated temperatures are predicted to directly impact river flows. A recent study estimated that for every one-degree Celsius rise in temperature, Colorado river flows will decline by 3–10% [18].

In this study, we explored the ways in which a new era of forest management, including accelerating the pace and scale of forest thinning, could be used to address declining conditions in forests in central and northern Arizona. At about 6,000 hectares per year (15,000 acres/year), the current pace of mechanical thinning in these forests does not begin to match the magnitude of recent forest disturbances. However, a new congressionally funded program called the Four Forest Restoration Initiative (hereafter “4FRI”) will accelerate the use of mechanical thinning and prescribed burns across four national forests, treating 238,000 ha (588,000 acres) in the first analysis area over the next 10 years. The objective of the 4FRI project is “…to re-establish forest structure, pattern, and composition, which would lead to increases in forest resiliency and function,” including reductions in fire risk and improved watershed function [19]. Mechanical thinning alone or in combination with prescribed fires reduced fire risk, increased runoff, and improved tree water-stress at the plot scale [20]–[23]. The 4FRI project provided the opportunity to evaluate the effectiveness of forest thinning to improve forest resilience and ecosystem functioning at larger scales.

**Figure 1:**
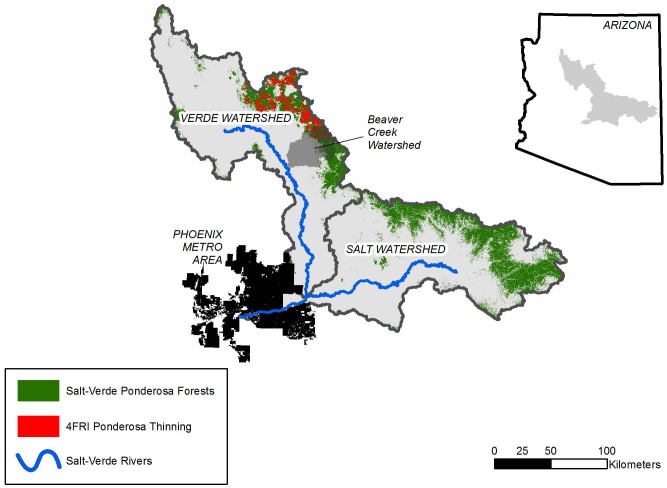
Map of Study Area. Map showing ponderosa pine forests in Salt-Verde watersheds in central Arizona, including those forests that are slated for mechanical thinning within the 4FRI project. Runoff from snowmelt in these forests is primary source of flow to Salt-Verde rivers which in turn are major sources of water for communities in the Phoenix Metro Area. Study used runoff model developed from experimental studies conducted in Beaver Creek watershed [20]. *Inset*: Location of study area in Arizona.

The objectives of this study were to: (1) investigate how variation in climate and the pace and extent of thinning of ponderosa pine forests affects runoff in the Salt and Verde watersheds of central Arizona; and (2) explore the management implications of using accelerated thinning to improve forest resilience in a time of declining forest and water conditions. We chose the Salt-Verde watersheds because forests from these watersheds provide water for 1.5 million people in Phoenix, which is the 6th largest city in the United States. We focused on ponderosa pine because this forest type produces 50% of the runoff in these watersheds even though it accounts for only 20% of the area [24]. We modified a forest runoff model developed from historical paired watershed experiments in the Beaver Creek sub-watershed within our study area [20] and ran the model in multiple scenarios to estimate additional annual runoff from mechanical thinning. The scenarios simulated forest treatment at two scales (a) landscape-scale thinning planned within the first analysis area of the 4FRI project and (b) watershed-scale thinning in forests across the Salt-Verde watersheds (Figure 1). Scenarios were designed to account for variability in winter precipitation, as well as the pace and extent of forest treatments. We estimated runoff in periods with below-average precipitation, herein referred to as droughts, and in periods with above-average precipitation, herein referred to as pluvials, using a model of 20^th^ century precipitation in ponderosa pine forests in the region [25]. We believe this is the first attempt to estimate the influence of mechanical thinning on runoff over multi-year broad-scale restoration projects that accounts for the effects of climate variability.

## Materials and Methods

The subsequent sections provide detailed information on the study area, the development of the modified runoff model, the method we used to simulate variability in winter precipitation, the 4FRI runoff scenarios, and the Salt-Verde runoff scenarios. All statistical analyses and graphics were completed using the statistical software SigmaPlot 11 (Systat software Inc, San Jose, CA), except for regression model fitting which was performed using Matlab 2013 (Mathworks, Natick, MA). Methods and results are reported in SI units, but for accessibility to forest and water managers, we reported English units in parentheses within the text and included all study figures and tables in English units as File S1. Area and water volume amounts were rounded off to three significant figures.

### Study Area

Ponderosa pine forests occupy approximately 0.68 million ha (1.68 million acres) of the 3.46 million ha (8.56 million acres) within the Salt-Verde watersheds in central Arizona (Figure 1). A portion of these forests, all within the Verde watershed, will be mechanically thinned in the 4FRI project in the next 10 years. Within the Salt-Verde watersheds, ponderosa pine forests grow at mid-elevations, from 1,800 to 2,600 m (5,900–8,600 feet) [26], [27], on soils derived from basalt (59%) and sedimentary rock (41%) [28]. Mean annual precipitation for ponderosa forests in the southwestern United States ranges from 510 to 760 mm (20 to 30 inches) and is highly variable [29]. Sixty percent of annual precipitation falls primarily as snow in the winter and spring months from October to April, and the melting of the resulting snowpack accounts for 80 to 90% of annual stream flow [29].

### Forest Treatment Runoff Model

For the purposes of this study, we were interested in estimating the additional runoff that becomes available due to forest thinning on an annual basis, not absolute values of watershed runoff. We define “additional runoff due to thinning” as the portion of precipitation that appears as surface water at the sub-watershed outlet and that is directly attributable to mechanical thinning treatments. This additional runoff can be considered as “in-place” or “in-situ” as we did not attempt to model runoff accumulation, routing, groundwater outflows or inflows, or channel losses in downstream watersheds.

We modified the Baker-Kovner logistical regression equation from the historical Beaver Creek sub-watershed experiments to calculate annual runoff in our study [20]. We selected this model because the Beaver Creek sub-watershed is actually within our study area (Figure 1), and because the model was based upon measured empirical data. These experiments and others like it demonstrated that reductions in forest basal area of 30–100% in ponderosa forests on basalt-derived soils increased runoff between 15–40% for up to six years after the initial treatment [29]. Runoff increases were negligible for basal area reductions below 30%, six years after treatments, and for years with winter precipitation below 230 mm (9 inches) [20], [29]. The original model predicted annual watershed runoff based on three independent variables: winter precipitation (Oct-Apr), forest basal area, and a solar insolation index, and explained a fair amount of variability in total watershed runoff [20].

**Figure 2:**
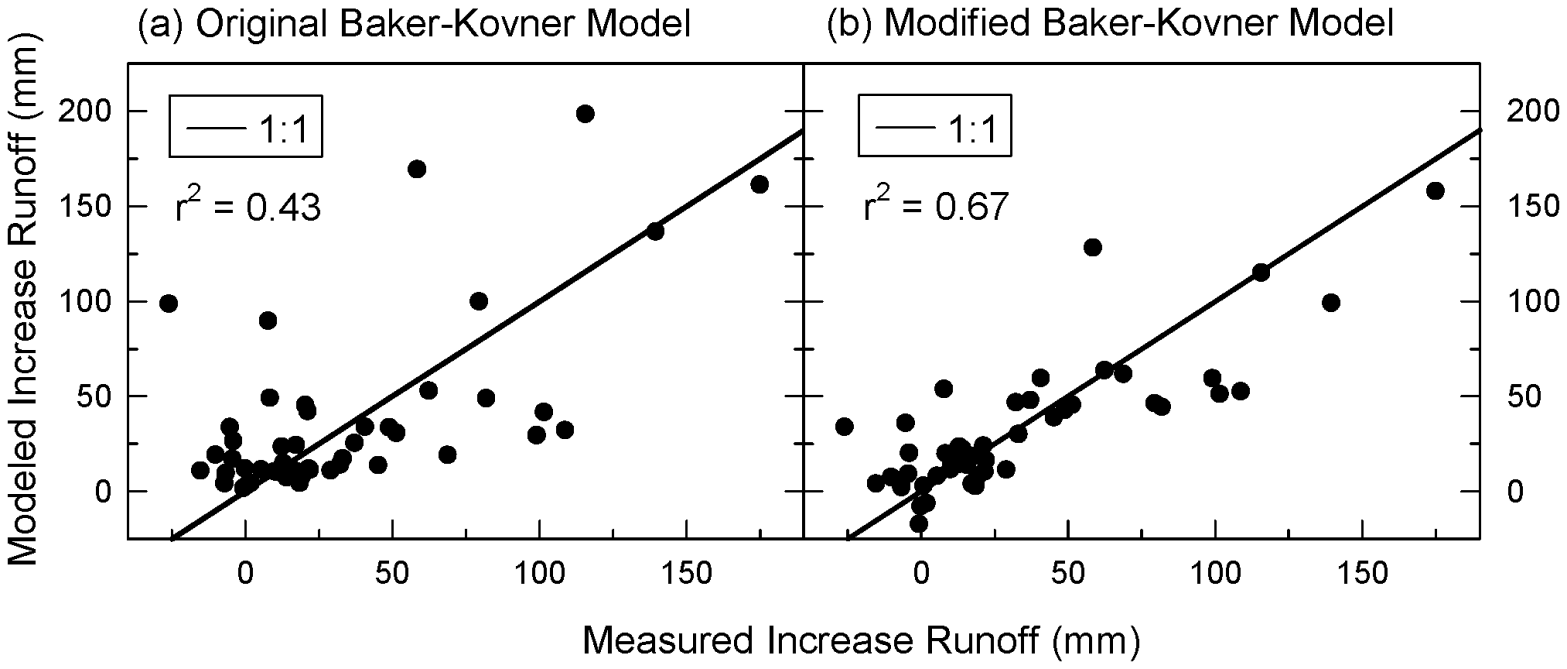
Comparison of models to observed runoff. Fit of (a) original [20] and (b) modified Baker-Kovner regression model output to increases in runoff associated with forests treatments in central Arizona, from Beaver Creek [30],[31] and Castle Creek [32] watersheds.

To derive increases in runoff directly associated with thinning using the paired watershed method, researchers in the Beaver Creek experiments first established a relationship between stream flows in the control and treated watersheds before treatments [30]. This relationship was used to predict what would have been baseline flows in the treated watershed. The difference between measured flows in the treated watershed and this predicted value were attributed to thinning effects. We compared data calculated in this fashion in the Beaver Creek experiments [30], [31] and one additional nearby site, Castle Creek [32], to output generated by the Baker-Kovner model. The output from the model was calculated as the difference in post-treatment and pre-treatment watershed runoff using post- and pre-treatment basal areas respectively and holding all other variables constant. In this comparison, we found the fit of the model output to the portion of runoff due to thinning to be much poorer (r^2^ = 0.43, Figure 2a) than model fit to total watershed runoff (r^2^ = 0.69). We concluded that the original regression model was relatively insensitive to the direct effects of forest treatments, including time since treatment, on runoff.

We used a stepwise regression procedure to develop a revised model, beginning with an initial model that included the independent variables (winter precipitation and basal area) that were found to be significant in historical experiments [20]. Input data for all independent variables used to develop the model were derived from empirical data collected during the Beaver Creek and Castle Creek historical experiments and archived in databases or published reports [30]–[32]. We added independent variables and used an F-test to test the null hypothesis that the coefficient of each term was zero at the 95% significance level. To ensure that the model was not over-fitted, we repeated the stepwise regression procedure with 70% of the original data, selected randomly. We determined that the structure of the model did not change and provided a good fit to the remaining 30% of the data. We examined the residuals from the model and found no discernible patterns of dispersion or bias. Including terms for slope, percent of watershed area with a south-facing aspect, and mean, minimum, and maximum winter temperatures did not significantly improve the model.

The final model included three terms: winter precipitation, an interaction term between winter precipitation and years since treatment, and an interaction term between winter precipitation and basal area. It can be expressed as: 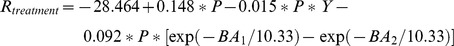



r^2^ = 0.67

where

*R_treatment_* = Increase in Annual Runoff attributed to Forest Thinning in mm

*P* = Total Winter Precipitation (Oct-Apr) in mm

*Y* = Years since Treatment

*BA_1_* = Basal Area before Treatment in m^2^/ha

*BA_2_* = Basal Area after Treatment in m^2^/ha

**Figure 3:**
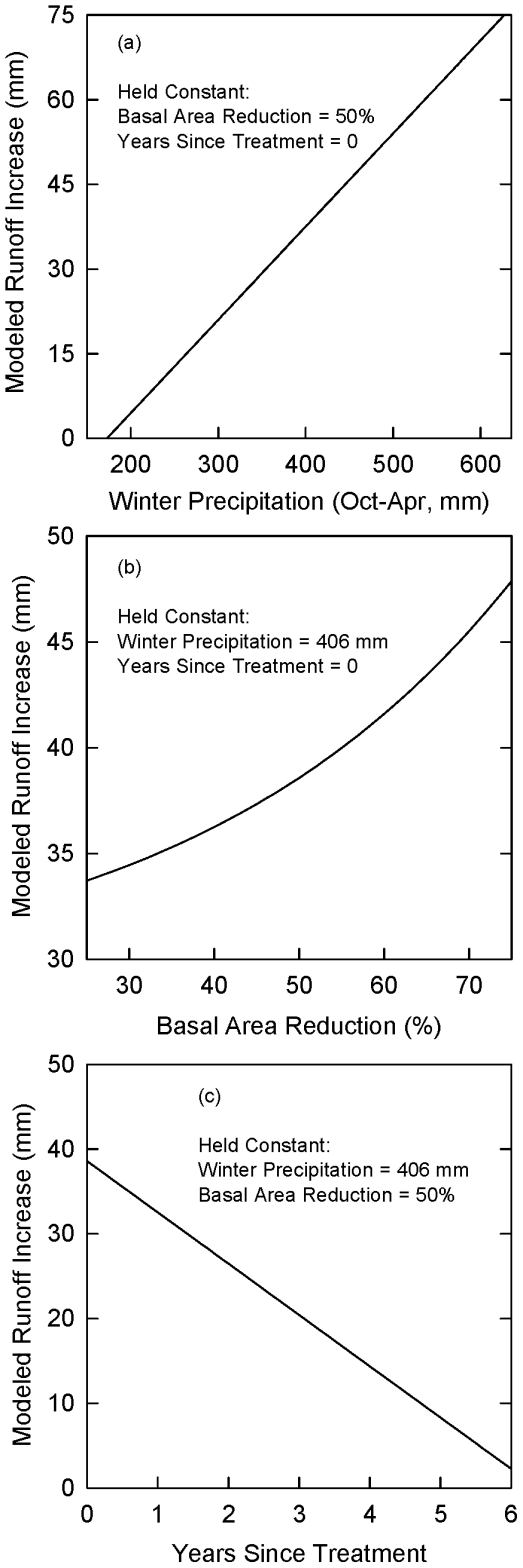
Influence of independent variables on runoff model. Relationships between model output to values for independent variables, including (a) winter precipitation, (b) percent basal area reduction, and (c) years since treatment. In all cases, other independent variables are held constant in order to view relationship of independent variable plotted on X-axis to model output.

Figure 2b shows that the revised model explained more of the variability in predicting additional runoff associated with forest treatments (r^2^ = 0.67) than did the original model. The model predicted runoff increases in a linear fashion with winter precipitation and basal area reductions, and decreases linearly with years since treatment (Figure 3). To compare the original and modified Baker-Kovner models in English Units, see File S2.

We did use the original Baker-Kovner regression equation in this study to calculate total watershed runoff for forests in their current condition so that we could estimate the percentage increase in runoff associated with treatments. For these calculations, we used the average value for the solar insolation index, r = 0.7, reported from the Beaver Creek experiments [20]. In a sensitivity analysis, we found that calculating absolute runoff based on the low (r = 0.66) and high values (r = 0.74) of this index changed the estimated increase from treatments by only 1–2 percentage points.

### Winter Precipitation

**Figure 4:**
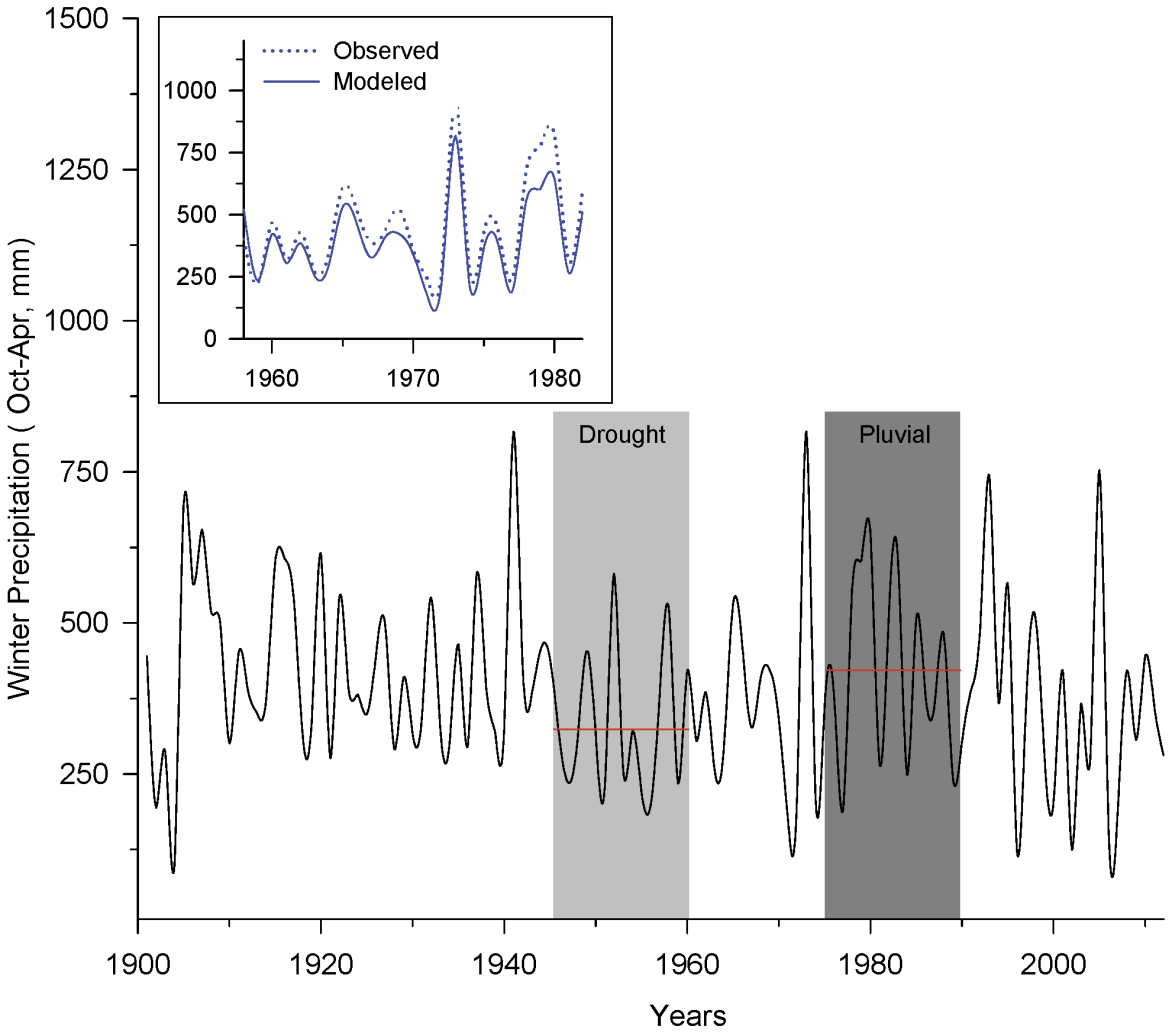
Variability in winter precipitation in ponderosa pine forests. Estimates of historical winter precipitation from 1900–2012 in ponderosa pine forests within Verde watershed from PRISM model [25]. Shaded areas are examples of 15-year droughts and pluvials that were used in study scenarios; horizontal red lines represent mean winter precipitation within these shaded areas. *Inset*: Comparison of measured winter precipitation observed during the historical Beaver Creek watershed experiments [20] from 1958–1982 versus modeled winter precipitation data shown in main figure.

In order to account for inter-annual and decadal variability of winter precipitation, we used the PRISM model [25] to extract pluvials and droughts from the 20^th^ century and inserted levels of winter precipitation from these periods into our runoff scenarios. We first extracted total winter precipitation, summed across October to April, for every year from 1900–2012 from the PRISM modeled dataset [25] across 2 scales: (a) ponderosa pine forests within the Verde watershed and (b) these forests within the Salt-Verde watersheds. We selected these geographies to be most representative of conditions for the 4FRI and Salt-Verde runoff scenarios, respectively. From 1900–2012, mean winter precipitation in Verde ponderosa pine forests was 394 mm (15.5 inches) with a range of 99–815 mm (3.9–32.1 inches) (Figure 4). For Salt-Verde forests, the mean was 368 mm (14.5 inches) and the range was 89–747 mm (3.5–29.4 inches) for this same period of time.

To test the accuracy of these modeled data, we compared values from the Verde ponderosa pine PRISM modeled data to measurements of total winter precipitation recorded during the original Beaver Creek experiments [31]. Given that we found the distribution of the data to be non-normal, we ran the non-parametric Spearman's rank order correlation. We found a strong association between modeled and observed data (Rho = 0.9577, p<0.001) although the model underestimated measured winter precipitation over the time period by approximately 25 mm (1 inch) and consistently under-estimated precipitation in wet winters (Figure 4 inset).

We calculated mean winter precipitation across 15-, 25-, and 35-year periods from this modeled data and used this information to select droughts and pluvials. For each scenario, we moved the start and end years that thinning was simulated to occur. For example, time periods for our 15-year scenarios included 1944–1958, 1945–1959, and 1946–1960 (examples illustrated as shaded areas in Figure 4). Using this method, we found two instances of pluvials – early 20^th^ century and late 1970s to late-1990s – and two droughts – during the 1950s and the current drought.

### Estimating runoff from 4FRI project

We used the modified regression model to calculate additional annual runoff from individual forests stands that will be thinned in the first analysis area of the 4FRI project, established an annual thinning schedule, and then summed increases in runoff from individual stands across treatment years. We ran a total of 26 scenarios where in each scenario we inserted a different 15-year winter precipitation sequence.

**Figure 5:**
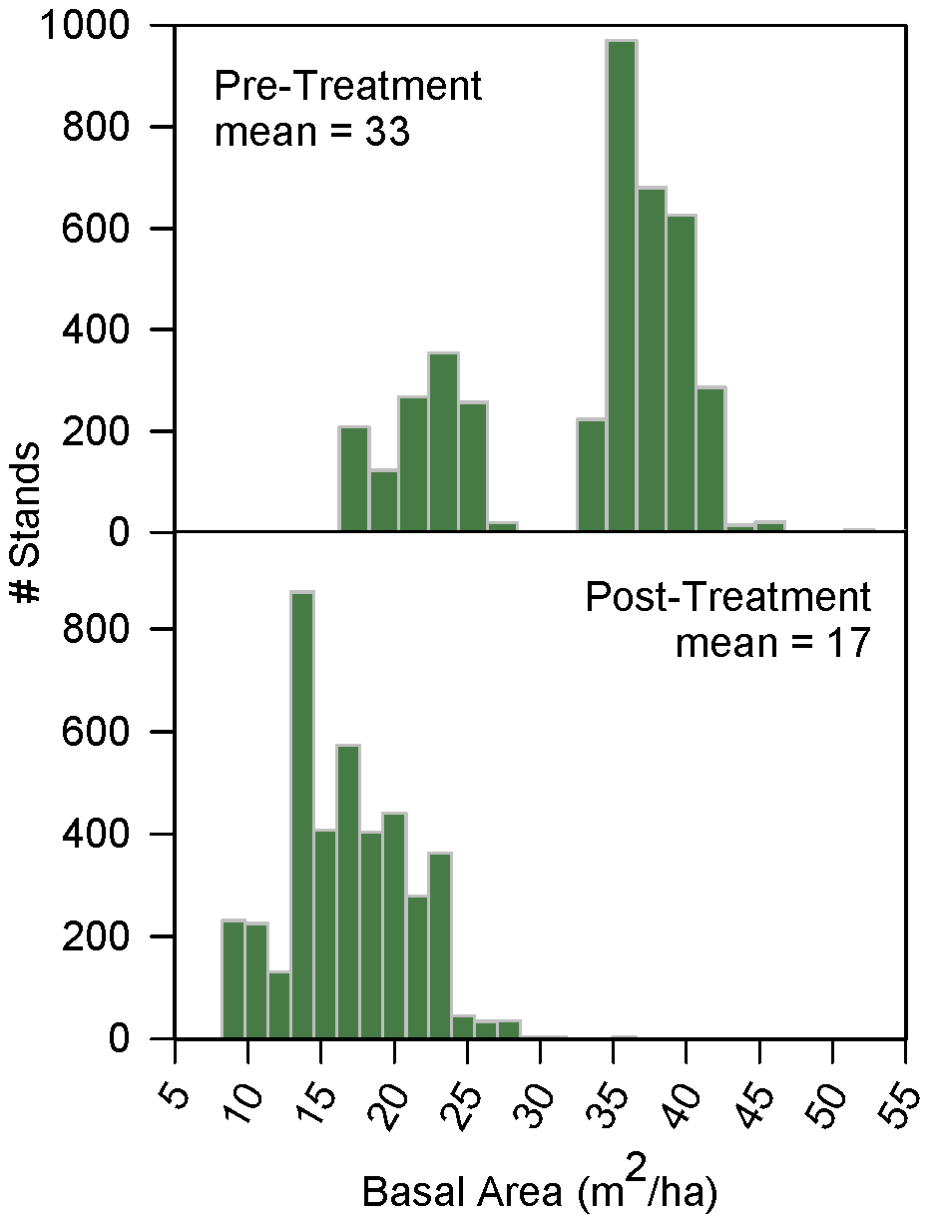
Ponderosa pine basal area reductions in 4FRI project. Histograms showing (top) pre-thinning and (bottom) desired post-thinning basal areas (in m^2^/ha) of ponderosa pine stands in the first analysis area of the 4FRI project (excluding stands where basal area reduction < = 30%).

We obtained a Geographic Information System (GIS) dataset of alternative C in the draft environmental impact statement for the first analysis area of the 4FRI project [19]. For each stand to be mechanically thinned, this dataset contained estimates of current basal areas and desired post-treatment basal areas. Forest prescriptions for “group-selection” stands called for two post-treatment basal areas, one for “open” areas that would be evenly thinned and another for “group” areas where thinning would be minimal to enhance wildlife habitats by retaining greater tree densities. For these stands, we calculated a post-treatment basal area that was an average of these two basal areas, weighted by the proportional area of the open and group areas. We selected 4,064 ponderosa pine stands within the Verde watershed slated for mechanical thinning, where the prescription will reduce basal areas by at least 30%. They ranged in size from 0.4 to 217 ha (1 to 536 acres), for a total of 61,900 ha (153,000 acres). The prescriptions within these stands called for reductions of basal area between 30% and 70% with a mean reduction of 48% (Figure 5).

**Figure 6:**
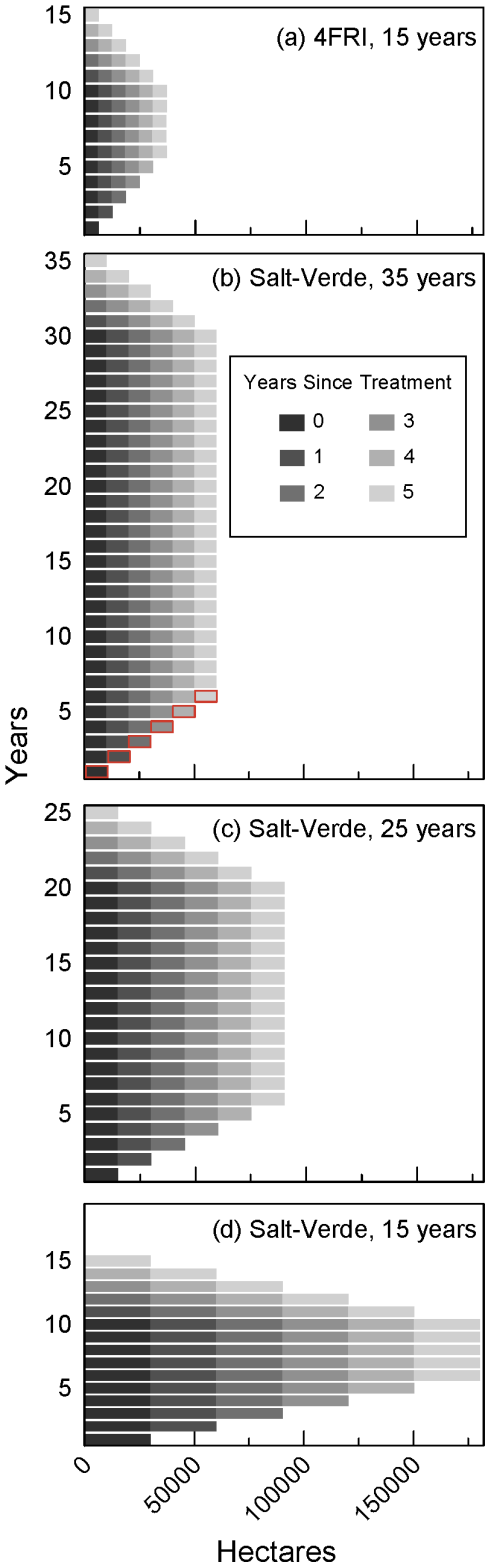
Forest treatment schedules for study scenarios. Graphical depiction of mechanical thinning treatment schedules for (a) 15-year 4FRI scenarios and (b-d) 35-year, 25-year, and 15-year Salt-Verde moderate thinning scenarios (total thinned area was 301,000 ha or 743,000 acres). Scenarios assumed consecutive treatments for 10-, 20-, and 30-year treatment periods shown as black bars in the bottom left portion of each of the figures. Bars outlined in red in (b) show the contribution of one cohort of stands through six years in the scenario.

Based on consultation with US Forest Service staff, we found that it was reasonable to assume that an equal number of hectares will be mechanically thinned every year across the 10-year treatment period. So for the purposes of our 4FRI scenarios, we assigned each of these stands to one of ten cohorts until the area of each cohort equaled 1/10th of total area or approximately 6,190 ha (15,300 acres). Figure 6a illustrates the treatment schedule for the 15-year 4FRI scenarios reported in this study. Cohorts were treated consecutively in the first 10 years and it was assumed that cohorts influenced runoff for 6 years. So, for example, stands treated in cohort 1, contributed to additional runoff in scenario years 1-6 and stands treated in cohort 10 contributed from years 10–15.

We calculated additional runoff associated with thinning and total watershed runoff by inserting values of the independent variables into the revised and original Baker-Kovner regression models, respectively. Pre-treatment and post-treatment basal area values were derived from the 4FRI stand data. Years since treatment ranged from y = 0 to 5. As described previously, winter precipitation values were droughts and pluvials drawn from the PRISM model for ponderosa forests in the Verde watershed. We set runoff increase to zero in years when winter precipitation was less than 230 mm (9 inches). We transformed the unit of runoff from mm to volume (million m^3^) using the area of each stand. We estimated annual increases in runoff at the landscape scale by summing stand level amounts for each scenario year, and calculated summary statistics (mean, median, max, cumulative) that allowed for a comparison of scenarios.

### Estimating runoff from Salt-Verde watersheds

Unlike the first analysis area of the 4FRI project, there was not a planned forest restoration project across the larger geography of the Salt-Verde watersheds. We developed a range of estimates for the extent, pace, and intensity of forest thinning that could be conducted over this larger geography, grouped these estimates into runoff scenarios, and ran the scenarios using the revised and original regression models to estimate additional runoff from treatments and total watershed runoff. To estimate the potential areal extent of thinning, we subtracted from the total forested areas those land uses that are typically considered unsuitable for mechanical thinning. We bracketed this initial area estimate with lower values to account for the lack of comprehensive spatial information. Assuming that the pace of treatments at this scale could take longer, we constructed 15-, 25-, and 35-year runoff scenarios. Finally, we assumed that the intensity of forest thinning at this scale would be similar to the range of basal area reductions planned for the first analysis are of the 4FRI project. The remaining parts of this section describe these steps in greater detail.

Based on a methodology developed in a previous study that estimated wood supply on four National Forests in northern Arizona [33], we subtracted from the total area of ponderosa pine in the watersheds – 0.68 million ha (1.68 million acres) – those areas that are typically excluded from mechanical thinning projects due to steepness, restrictions in land management, previous treatments, or considerations of soil, habitat, or wildlife conditions (Table 1). We adopted 6 of the 7 “exclusion” criteria from that study and added one additional factor. We removed one criterion – Northern goshawk nest areas –because the mean basal area reduction for these areas in the 4FRI project was greater than 30% [19] suggesting that thinning in these areas could result in additional runoff based on the results of the Beaver Creek experiments [20]. We added one criterion – high severity burn patches – because a nearby study on the recovery of ponderosa pine forests after wildfire demonstrated that mature forests suitable for thinning would not likely develop in areas that had burned with high severity within the timeframe of this study [34]. While two of the three 4FRI prescriptions for Mexican Spotted Owl Protected Activity Centers (MSO PAC) would result in negligible changes in basal area, one would allow for basal area reductions greater than 30% [19]. However, we were not able to differentiate among these prescriptions from the data we obtained, so we opted for a conservative estimate for this factor by excluding MSO PAC areas from our analysis. Compiling the publicly available geospatial data for these factors [26], [27], [33], [35]–[38], we found that 401,000 ha (992,000 acres), or 59% of ponderosa pine forests, were available for mechanical thinning after accounting for these exclusion areas. The percentage of forests excluded in this estimate – 41% – compared favorably with the average of 37% of forests that were excluded in dozens of restoration projects evaluated in the northern Arizona [33]. We bracketed this high estimate with a moderate estimate – 45% or 301,000 ha (743,000 acres) – and a low estimate – 30% or 204,000 ha (505,000 acres) – because quantitative geo-spatial data on some exclusion areas was missing or incomplete. As illustrated graphically for the moderate estimate in Figure 6b-d, we divided these areas into 10-, 20-, and 30-year treatment cohorts assuming that equal areas would be thinned every year.

**Table 1:** Estimate of hectares of ponderosa pine forests available to mechanical thinning in Salt-Verde watersheds.

Category		Hectares	%	*Sources*
*Salt-Verde Watersheds Ponderosa Pine Forests*		*681,000*	*-*	[27]
*Exclusions*				
	*Steep Slopes* greater than or equal to 40%	68,000	10%	[26]
	*Hi-Severity Burn Patches* within Wallow and Rodeo-Chedeski Fires	43,700	6%	[35]
	*Specially Designated Areas* including Wilderness Areas, Research Natural Areas, State Wildlife Management Areas^a^	21,800	3%	[36],[37]
	*Streamside Management Zones* within 100 feet perennial reaches in national forests and 200 feet in tribal areas	8,050	1%	[38]
	*Mexican Spotted Owl Protected Activity Centers* where owls have been found to be nesting^b^	57,500	8%	[33]
	*Erodible Soils* where thinning is unsuitable due to erosion risk or rocky conditions^c^	93,100	14%	[33],[37]
	*Completed Treatments* that have been thinned in last 10 years^d^	35,200	5%	[37]
*Sub-Total Exclusions (accounting for overlap between layers)*		*280,000*	*41%*	
*Sub-Total Forest Available Mechanical Thinning*		*401,000*	*59%*	

a Estimate derived from USDA Forest Service Southwestern Region Wilderness Status layer and Forest Level Special Interest Management Areas layers [37].

b Data available for Coconino, Apache-Sitgreaves, Kaibab, and Tonto National Forests only. Added qualitative estimate for this exclusion factor for tribal lands and Prescott National Forest assuming land excluded in these land units would be proportional to lands excluded for this factor on the four national forests above.

c List of excluded soils for Coconino, Apache-Sitgreaves, Kaibab, and Tonto National Forests based on Hampton et al. [33]. This list of excluded soils was augmented based on consultation with USDA Forest Service southwestern region soil scientists. Geospatial data for these soils derived from USDA Forest Service Forest Level Terrestrial Ecological Units layers [37]. Added qualitative estimate for this exclusion factor for tribal lands and Prescott National Forest where geospatial data was unavailable, assuming land excluded in these land units would be proportional to lands excluded for this factor on the four national forests above.

d Geospatial data for this factor derived from USDA Forest Service Forest Level Activities Layer joined to FACTs table for Coconino, Apache-Sitgreaves, Kaibab, Tonto, and Prescott National Forests [37]. Added qualitative estimate for this exclusion factor for tribal lands based on consultation with Bureau of Indian Affairs forestry personnel.

Summary of hectares and percentages of ponderosa pine forests within Salt-Verde watersheds in relation to potential for thinning treatments, including total hectares, those areas typically excluded from mechanical thinning, and the remaining hectares available for mechanical thinning. Estimates rounded off to three significant digits.

We scaled up the forest condition information contained in the 4FRI stand data using a proportion metric, assuming that these stands were representative of stand-level forest conditions across the Salt-Verde watersheds. For example, 4FRI stands that comprised 1,500-ha in a 15,000-ha 4FRI treatment cohort were estimated to be 3,000-ha in a 30,000-ha Salt-Verde cohort. If the thinning prescription for these stands called for a reduction in basal area from 33 to 17 m^2^/ha, then the Salt-Verde scenarios calculated additional runoff from 3,000-ha that are thinned from 33 to 17 m^2^/ha. After scaling up the area of each stand to the watershed scale, the procedure for estimating runoff in the Salt-Verde scenarios was the same as described for the 4FRI scenarios. We ran 147 scenarios in total, 67 fifteen-year, 52 twenty-five year, and 28 thirty-five year scenarios.

## Results

### Four Forest Restoration Initiative Runoff Scenarios

**Figure 7:**
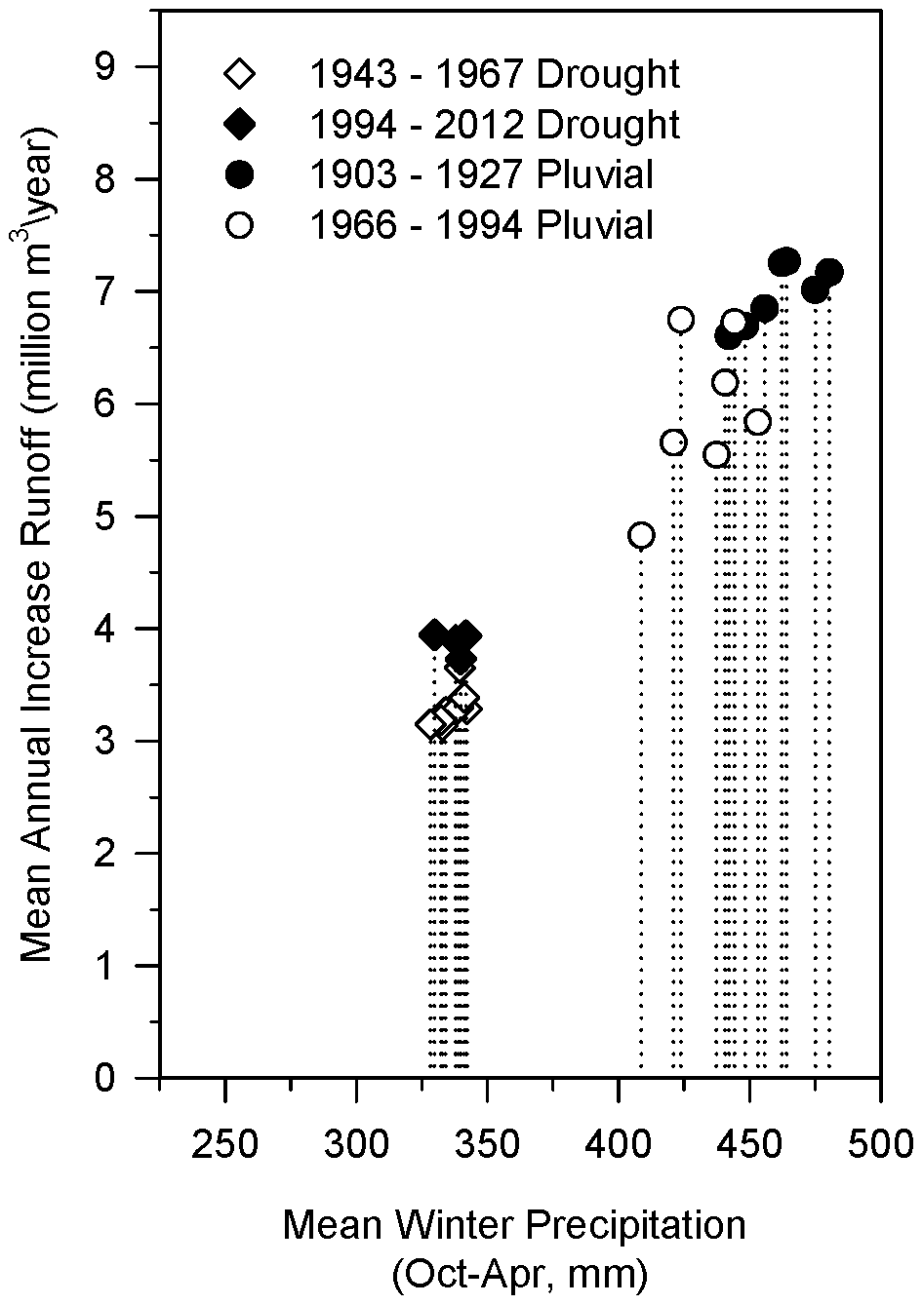
Increases in mean annual runoff from thinning in 4FRI project. Results from 26 scenarios with varying levels of winter precipitation showing increases in mean annual runoff associated with mechanical thinning of ponderosa pine forests in the first analysis area of the 4FRI project. In order to compare scenarios, only increases in *mean* annual runoff are shown. Annual variability in runoff for two of these scenarios is shown in Figure 8.

Mechanical thinning of ponderosa pine forests in the first analysis area of the 4FRI project – 6,190 ha/year for ten years totaling 61,900 ha (15,300 ac/year; 153,000 acres total) – increased mean annual runoff from 3.13 million m^3^ (2,540 acre-feet) in a simulated drought to 7.27 million m^3^ (5,890 acre-feet) in a pluvial. Differences in winter precipitation over the 15-year simulation periods significantly influenced runoff gains. A difference in mean winter precipitation of only 130 mm (5 inches), from 330 mm (13 inches) in drought scenario to 460 mm (18 inches) in a pluvial scenario, resulted in a doubling of the annual increase in runoff from treatments (Figure 7).

**Figure 8:**
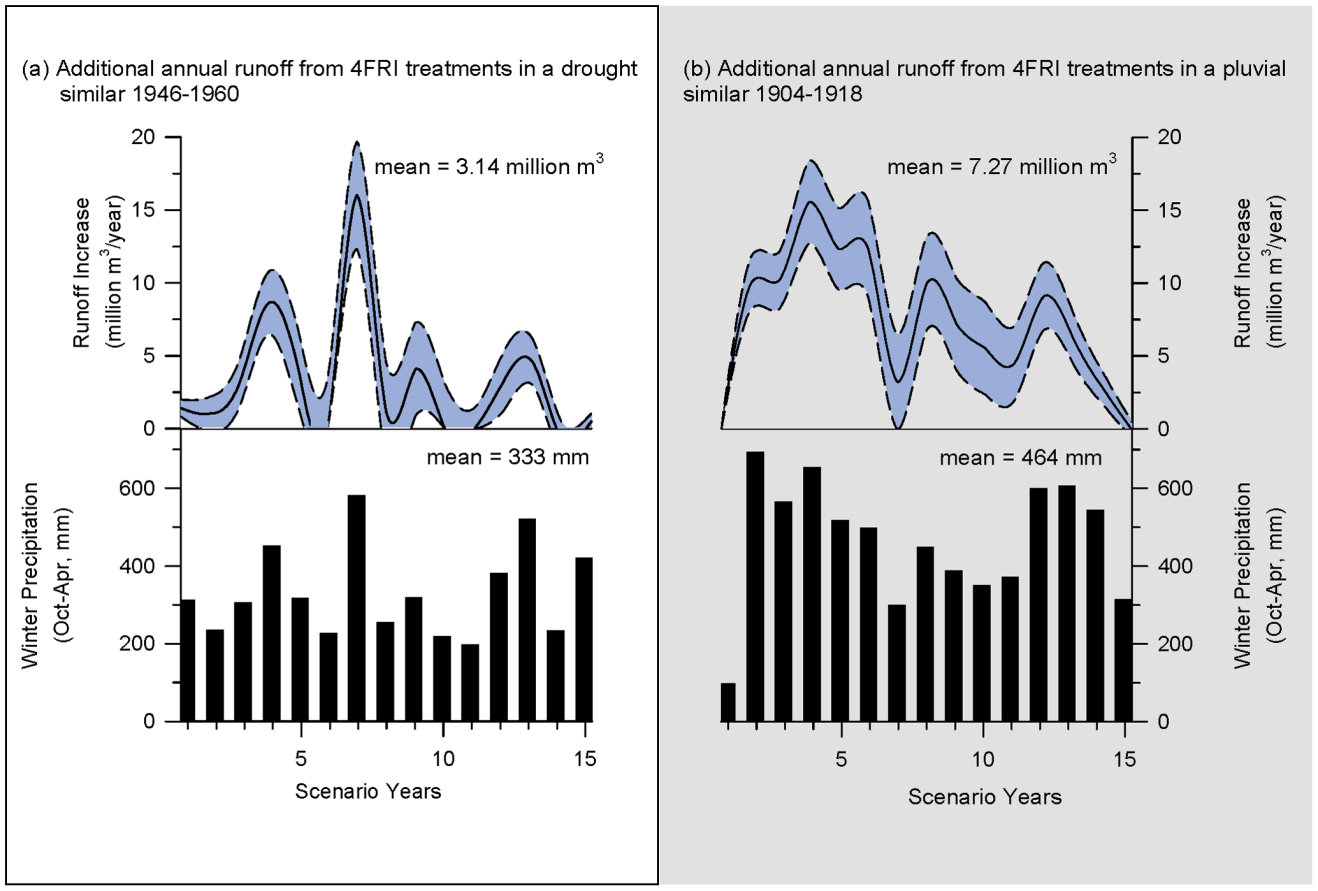
Year to year variability in runoff increases from thinning in 4FRI project. Modeled increases in annual runoff associated with mechanical thinning of ponderosa pine forests in the first analysis area of the 4FRI project during the (a) drought and (b) pluvial that produced lowest and highest gains in runoff respectively. Top panes show increases in annual runoff in million m^3^/year. Solid black lines are output values from regression model; dotted lines and blue areas represent 90% confidence intervals. Bottom panes show corresponding winter precipitation values (Oct-Apr, mm) used as one of the independent variables to calculate runoff.

**Figure 9:**
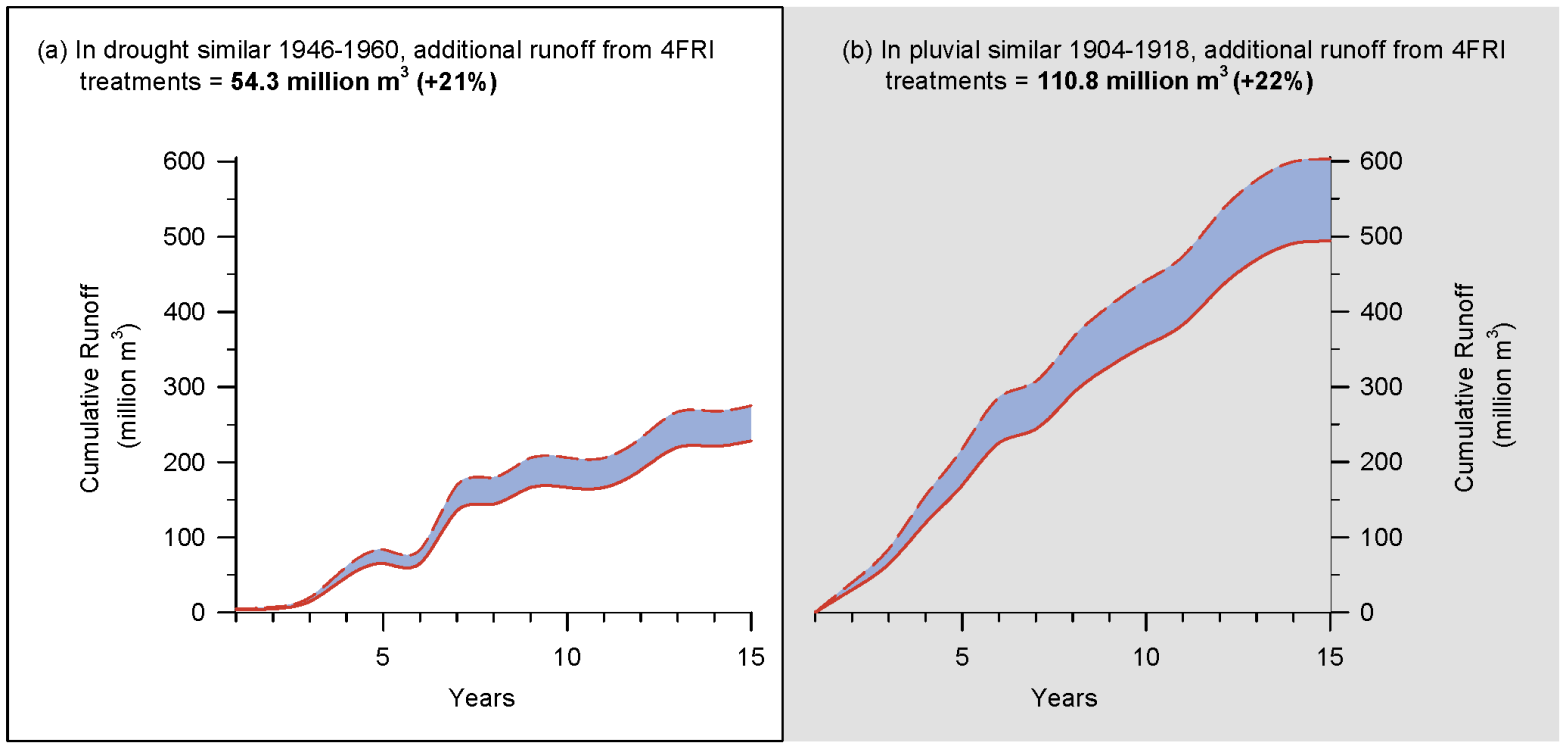
Cumulative runoff increases from thinning in 4FRI project. Estimates of cumulative increases in runoff (million m^3^) from planned mechanical thinning of ponderosa pine forests in the first analysis area of the 4FRI project under (a) drought and (b) pluvial conditions. Solid red lines are estimates of cumulative runoff under current forest conditions using original Baker-Kovner regression model [20]. Dotted red lines represent increases in cumulative runoff associated with 4FRI treatments using modified Baker-Kovner regression model. Difference between these two values, shown with blue shading, is additional runoff from forest thinning treatments. Estimated increases in runoff ceased after 15 years.

Inter-annual variability in winter precipitation was also important. Figure 8 illustrates year to year increases in runoff for those scenarios that resulted in the lowest and highest gains in runoff. Years with high winter precipitation played a disproportionate role in additional runoff in both droughts and pluvials. In the majority of scenarios ran (15 out of 26), winter precipitation from only 5 of the 15 years accounted for at least 75% of the increased runoff. Cumulative increases in runoff across 15-year periods resulted in a total increase from 54.3 to 111 million m^3^ (44,000–89,800 acre-feet) (Figure 9). Runoff from thinned forests was approximately 20% greater than unthinned forests (as estimated using original Baker-Kovner regression model) in both droughts and pluvials (data not shown).

### Salt-Verde Runoff Scenarios

**Figure 10:**
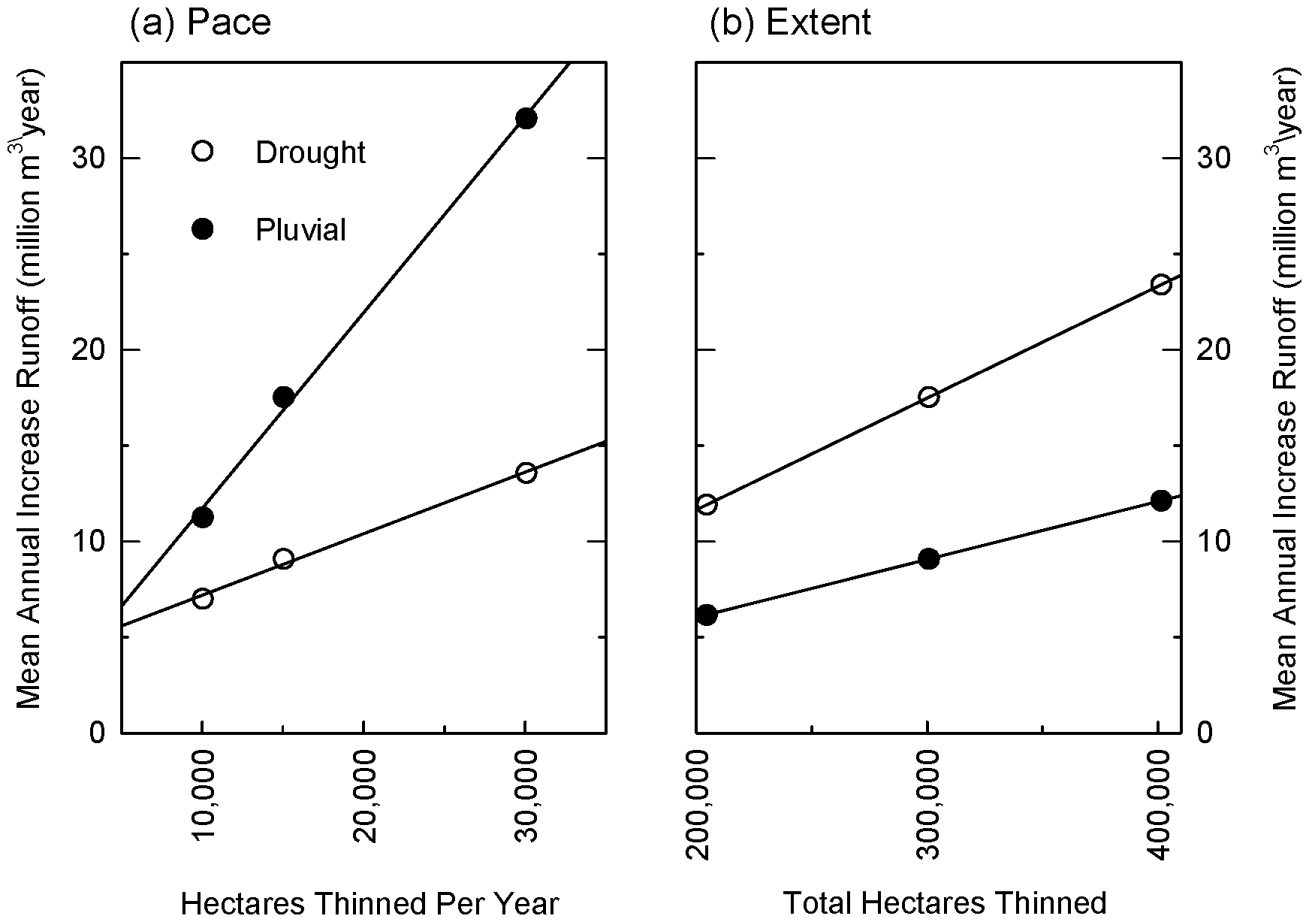
Scale effects of thinning on runoff in Salt-Verde watersheds. Effects of increasing (a) pace and (b) extent of thinning treatments in ponderosa pine forests in Salt-Verde watersheds on increases in mean annual runoff (million m^3^/year). In (a) total area thinned is held constant at 301,000 ha (743,000 acres) (scenarios: 35mid, 25mid, 15mid) to show influence of increasing the area thinned per year. In (b) duration of thinning treatments is held constant at 25 years (scenarios: 25low, 25mid, 25high) to show influence of increasing the total area thinned across the scenario. In order to illustrate scale effects, only increases in *mean* annual runoff are shown. Statistics describing annual variability in runoff gains are shown in Table 2 and illustrated graphically for 4FRI scenario in Figure 8.

Depending on winter precipitation and the forest treatment schedule, mean annual increases in runoff from thinning of ponderosa forests across the Salt-Verde watersheds ranged from 4.76 to 15.0 million m^3^ (3,860–12,200 acre-feet) over a 35-year treatment period, 6.18 to 23.4 million m^3^ (5,010 to 19,000 acre-feet) over 25 years, and 9.23 to 42.8 million m^3^ (7,480 to 34,700 acre-feet) over 15 years (Table 2). Similar to the 4FRI scenarios, additional runoff in the Salt-Verde watersheds was 1.6–2.3 times greater in pluvials than in droughts. Regardless of whether the scenarios occurred in a drought or pluvial, cumulative runoff gains in thinned forests were 20-26% greater than unthinned forests. Cumulative gains ranged from 167 to 525 million m^3^ (135,000–426,000 acre-feet) in the 35-year scenarios, 154 to 585 million m^3^ (125,000–474,000 acre-feet) in 25-year scenarios, and 138 to 643 million m^3^ (112,000–521,000 acre-feet) in 15-year scenarios. In both droughts and pluvials, runoff increased in a positive linear fashion with increases in the pace and the extent of forest thinning (Figure 10). See File S1 to see study figures and tables in English units.

**Table 2:** Annual runoff increases from thinning in Salt-Verde watersheds.

Forest Management: Thinning^a^				Winter Precipitation Regime			Runoff: Annual Increase			Runoff: Cumulative Increase	
*Scenario Name*	*Total Hectares*	*Hectares per Year*	*Effective Hectares per Year*	*Type*	*Similar to 20th Century Period*	*Mean (mm/yr)*	*Mean (million m^3^)*	*Median (million m^3^)*	*Maximum (million m^3^)*	*Total (million m^3^)*	*%*
35-low	204,000	6,800	35,000	Drought	1942–1976	340	4.76	2.99	17.5	167	23%
				Pluvial	1965–1999	396	7.65	5.85	25.7	268	26%
35-mid	301,000	10,000	51,400	Drought	1942–1976	340	7.01	4.39	25.8	245	23%
				Pluvial	1965–1999	396	11.3	8.61	37.7	393	26%
35-high	401,000	13,400	68,800	Drought	1942–1976	340	9.35	5.86	34.4	327	23%
				Pluvial	1965–1999	396	15.0	11.5	50.4	525	26%
25-low	204,000	10,200	49,000	Drought	1942–1966	338	6.18	4.06	26.3	154	20%
				Pluvial	1975–1999	411	11.9	9.65	33.6	299	22%
25-mid	301,000	15,100	72,000	Drought	1942–1966	338	9.09	5.97	38.7	227	20%
				Pluvial	1975–1999	411	17.5	14.2	49.3	438	22%
25-high	401,000	20,100	96,300	Drought	1942–1966	338	12.1	7.98	51.7	303	20%
				Pluvial	1975–1999	411	23.4	19.0	66.0	585	22%
15-low	204,000	20,400	81,700	Drought	1945–1959	315	9.23	5.95	52.3	138	20%
				Pluvial	1975–1989	422	21.8	8.31	60.1	327	22%
15-mid	301,000	30,100	120,000	Drought	1945–1959	315	13.6	8.75	77.0	204	20%
				Pluvial	1975–1989	422	32.1	12.2	88.4	481	22%
15-high	401,000	40,100	161,000	Drought	1945–1959	315	18.1	11.7	103	271	20%
				Pluvial	1975–1989	422	42.8	16.3	118	643	22%

a ‘Scenario Name’ indicates number of years simulated in each scenario (15, 25, 35 years) and extent of forest thinned (low = 30% of forest thinned; mid = 45%; high = 60%). ‘Total Hectares’ are total number of hectares thinned in each scenario. ‘Hectares per Year’ are number of hectares thinned every year for 10 consecutive years (15-year scenarios), 20 consecutive years (25-year scenarios), and 30 consecutive years (35-year scenarios). ‘Effective Hectares per Year’ are the average number of thinned hectares across all years of the scenario that contribute to additional runoff (see figure 6).

Estimates of additional runoff associated with thinning of ponderosa pine forests across Salt-Verde watersheds accounting for variation in treatment periods, total forest extent treated, and drought and pluvial periods of winter precipitation (Oct-Apr). Estimates rounded off to three significant digits.

## Discussion

This study demonstrated that the pace, extent, and intensity of forest thinning that is planned under the first analysis area of 4FRI and at larger scales could measurably increase runoff in ponderosa pine forests in central Arizona. Modeled runoff from thinned forests was approximately 20% greater than runoff from unthinned forests which is within the range of 10–40% increases in runoff demonstrated in the Beaver Creek experiments [20], [29]. The Salt-Verde runoff scenarios showed that levels of additional runoff increased proportionally with increases in the pace and extent of thinning. Runoff gains occurred in droughts and pluvials, a surprising outcome that underscored the importance of inter-annual and decadal variability in precipitation. These increases in runoff would likely improve conditions for water-dependent natural resources, such as cienegas, riparian areas, and aquatic habitats, which are vulnerable to low flows that are experienced seasonally, especially in summer months, and also during droughts [5]–[7]. Runoff gains also could provide incidental benefits to the water supply of downstream users, but the increases were more modest when compared to total runoff from the Salt-Verde watersheds. They would comprise a 0–3% increase from mean annual surface flows from the Salt-Verde rivers of 1.39 billion m^3^ (1.13 million acre-feet) and approximately a 1-9% increase of Salt-Verde flows supplied to municipal users in the Phoenix Metro Area on an annual basis [39].

Consistent with the historical experiments, thinning effects on runoff were temporary in this study. Additional runoff would cease six years after the multi-year thinning schedules in our scenarios. Beaver Creek investigators speculated that thinning effects were short-lived because of subsequent regrowth of understory species [29]. We note that prescribed fires are planned as subsequent treatments for all stands that are initially thinned within the first analysis area of 4FRI [19]. These maintenance treatments should provide additional information as to how effective subsequent actions are in terms of removing understory vegetation and sustaining runoff gains.

Our study adds to previous research on the potential for increases in runoff associated with thinning of ponderosa pine forests in the Southwest. Using a simulation model, Brown and Fogel [40] found that thinning approximately 9,300 ha (23,000 acres) of ponderosa pine per year over a 10-year period produced increases in runoff that ranged from 0.28 to 11.8 million m^3^/year (230–9,600 acre-feet/year). This range overlapped with but was lower than the 6.18 to 11.9 million m^3^/year (5,010–9,660 acre-feet/year) increases in runoff for the two Salt-Verde runoff scenarios with annual thinning of approximately 10,000 ha (25,000 acres) (Table 2). One potential reason for this difference was that Brown and Fogel [40] modeled the treatment-runoff relationship to be constant over time, 18 mm (0.71 inches), and did not evaluate variability in precipitation but instead assumed average winter precipitation of 396 mm (15.6 inches). Using similar values for winter precipitation and basal area reduction, our model simulated the runoff response to vary depending on years since treatment, from a high of 38 mm (1.5 inches) in the year immediately following treatments to a low approaching zero mm after six years (Figure 3c).

### Study Limitations

In this study, we revised a statistical runoff model from the historical Beaver Creek watershed experiments to explore how variations in climate and forest thinning would affect runoff. To the extent possible, we limited the application of the runoff model to the range of climatic and environmental conditions under which the original data were collected and model developed. However, our modeling approach was constrained by several uncertainties that will require further investigation. The model was limited by the scope of forest practices conducted in the Beaver Creek watershed experiments [20], [30]–[32]. These experiments used thinning techniques such as strip-thinning and patch clearing that had a similar range of basal area reductions but a different spatial configuration from contemporary thinning prescriptions. They did not measure the effects of maintenance treatments on runoff. They measured “in-place” runoff at sub-watershed outlets and did not measure other aspects of the water budget, such as evapotranspiration, soil moisture, surface routing, and groundwater recharge. The revised model represented statistical relationships between annual runoff and winter precipitation, forest basal area reductions and time since treatment, but these relationships were not necessarily related to physical processes or functions that control water balance [20]. We summarized how important factors that are not directly modeled may increase or decrease the range or runoff due to thinning that is documented in this study in File S3.

Of all the factors where uncertainties in forest hydrology and runoff remain, perhaps the most significant were the effects of soil types, long-term climate variability, and climate change. In terms of soil types, the revised regression model in this study was derived from experimental thinning on basalt-derived soils that support 59% of ponderosa pine forests in the Salt-Verde watersheds. The runoff effects of thinning on the remaining 41% of forests on sedimentary-derived soils are unknown. Two studies demonstrated that runoff from untreated ponderosa forests on sedimentary-derived soils can be substantially less than runoff from volcanic soils [41], [42]. Several properties of sedimentary-derived soils relative to basalt-derived soils may lead to greater water permeability and infiltration, including lower clay content, extensive fracturing, and deeper soil horizons [30], [43], [44]. Depending on a number of factors, increased infiltration may lead to increased recharge, which we would expect to result in increased discharge to surface water at some location downstream.

A nearby paired-watershed study in mixed conifer forests on sedimentary-derived soils found that overstory removal of 30-80% led to a slightly higher range of increased runoff, 30–110%, that persisted for more years than the Beaver Creek experiments [45],[46]. Direct comparisons with ponderosa pine forests are not possible because this study was conducted within a higher-elevation mixed-conifer forest that had higher initial basal areas and higher mean annual precipitation.

Relative to climate, this study's scenarios captured the high end of variability associated with pluvials but probably not the low end of variability associated with droughts. Specifically, the early 20^th^ century pluvial may have been the wettest pluvial that has occurred in the last 12 centuries [47], whereas the two occurrences of 20^th^ century drought were eclipsed in terms of duration and severity by several multi-decade “mega-droughts” [48]–[51]. Output from global circulation models indicates that climate variability will continue to be an important characteristic of the region in the future [52], but that climate change may increase the risk of extreme climatic events such as multi-decade droughts and extreme winter precipitation [53], [54]. Some global circulation models also project that mean winter precipitation in the Southwest will decline by up to 10% [52], but it may take many years to detect effects on stream flows because of precipitation variability [55]. The net effect of changes in precipitation on forest condition and hydrology still needs to be resolved and will require more investigation.

Unlike precipitation, the effects of anthropogenic warming on forest hydrology are clearer. Studies have found that warmer temperatures in recent decades help explain a downward trend in snowpack in the western United States, even after patterns of natural climate variability have been considered [9], [56]. In our revision of the historical runoff model, we attempted to add several parameters of temperature – seasonal means, maximums, and minimums – as explanatory variables to the revised regression model but none were significant. This suggests that the temporal and spatial range of winter temperatures that occurred during the years when the watershed experiments were conducted was not sufficient to add explanatory value to predicting runoff. In a separate trend analysis of winter temperatures (Oct-Apr), we found that temperatures in the months of March and April in the last 25 years, 1988–2012, were significantly warmer by 2 and 1 degrees Celsius (3.5 and 2 degrees Fahrenheit), respectively, than temperatures for the same months in the 25 years when the experiments were conducted, 1958–1982 (see File S4 for more detailed information). The magnitude of observed declines in snowpack in the Southwest, in the range of 20%, is similar to the increases in runoff associated with thinning from this study, suggesting that accelerated thinning may at least offset or ameliorate runoff losses due to climate change.

### Management Implications: Accelerated Forest Thinning to Improve Resilience

On balance, we believe there is enough evidence for managers to explore the ways in which accelerated forest thinning can restore forest resilience and functioning to ameliorate the drying of ponderosa pine forests associated with warming and past management activities. Below we summarize the management implications of this and related studies.

1. *Maintain & improve ecosystem function* – The current paradigm of forest management has moved away from a debate about managing forests to address one societal need, such as downstream water supply, to recognition that management strategies need to address multiple challenges [29], [57]. The 4FRI restoration project demonstrates this approach with planned benefits that include “…improved vegetation biodiversity, wildlife habitat, soil productivity, and watershed function” [19]. The impaired functioning and heightened vulnerability of southwestern forests to large-scale disturbances increases the urgency to improve ecosystem functioning, and to measure the effectiveness and trade-offs associated with these management activities using robust adaptive management and monitoring protocols. Such a focus may result in more options and flexibility in subsequent years, whereas failure to maintain functioning ecosystems may eliminate future options if soil and hydrologic processes are irreversibly altered.

Our study demonstrated that accelerated thinning can improve surface water runoff, a key ecosystem function, and provided indirect evidence of benefits for soil moisture and productivity. Another study found that average soil water content on low density ponderosa forests (250 trees/ha) was substantially higher than on high density forests (2,710 trees/ha), although these differences in forest density were not a result of mechanical thinning [58]. Researchers in the historical Beaver Creek experiments hypothesized that lower tree densities associated with thinning would reduce evapotranspiration losses, and thereby allow more water to be available for soil moisture, groundwater recharge, and surface water runoff [29]. Although we were unable to evaluate thinning effects on evapotranspiration, Dore et al. [59] found that light thinning (40% basal area reduction) in a ponderosa pine forest reduced stand-level evapotranspiration by 12% over four years, but the effect diminished over time and was not detectable in the fourth post-harvest year. This same study found that removal of all trees in a wildfire-burned site reduced annual evapotranspiration by 20%.

Other studies demonstrated that mechanical thinning can reduce the vulnerability of forests to uncharacteristic crown fires. Using a fire simulation model, Cochrane et al. [60] found that landscape-scale mechanical thinning that preceded actual wildfires could have reduced the average size of six wildfires in ponderosa pine and mixed conifer forests in California and the southwestern United States by an average of 18% (range 0.3 to 65%). A meta-analysis of 54 experimental studies showed that mechanical thinning significantly reduced the susceptibility of western ponderosa and Jeffrey pine forests to crown fires [23]. These studies describe the primary mechanism by which thinning reduces fire risk as a redistribution of fuels, thereby reducing fire spread rates and crowning behavior.

2. *Apply management tools at scale* – Managing forests at a scale that is comparable to recent forest disturbances will be another key factor for improving resilience. At 238,000 ha (588,000 acres) of ponderosa pine to be treated with mechanical thinning and prescribed fire, the 4FRI project is approaching the scale of these amplified disturbances. Our study demonstrated that potential increases in runoff associated with thinning were scale dependent: runoff increased in a linear fashion as the pace and extent of thinning increased in the Salt-Verde runoff scenarios (Figure 10). Recent efforts to reduce fire risk with landscape-scale treatments of ponderosa pine forests have been successful. For example, treatments completed before the largest wildfire in Arizona history, the Wallow Fire in 2011, effectively protected communities and towns by reducing fire severity before it reached key residential areas [61].

Further, accelerated thinning may open up opportunities to use other management tools at an accelerated pace. For example, wildland fire use has been an effective and inexpensive tool for maintaining forest resilience across large areas in remote forests and woodlands – the Gila Wilderness in New Mexico and Grand Canyon National Park in northern Arizona [62], [63] – but has been used less frequently in forests near population centers and towns. Completion of treatments in the first analysis area of the 4FRI project could have a multiplying effect on maintaining forest resilience if the thinning itself allows for increased use of wildland fire or prescribed fire over many more hectares.

*3*. *Leverage knowledge of climate variability* – The Beaver Creek watershed experiments and others like it demonstrated that winter precipitation was the best predictor of additional runoff from forest thinning [20], [30]. We add to this knowledge by examining how variability in winter precipitation could influence management effectiveness. Our scenarios demonstrated that gains in runoff from thinning could occur even under drought conditions. In simulated droughts, 1 out of 3 years had above-average precipitation, and these years accounted for 75% of the runoff gains in these scenarios. Even though these gains were substantially less than runoff gains during pluvials, additional runoff under drought conditions could help lessen the impact of predicted climate-driven losses in hydrologic connectivity important to native fish in the Salt and Verde watersheds [64].

This study also demonstrated that the same thinning schedule could result in a doubling in additional runoff if conducted in a pluvial typical of 20^th^ century variability as compared to a drought. Scientists have developed decision support tools to help managers understand the likelihood that precipitation regimes will shift from wet to dry conditions or vice-versa [65], [66]. Knowledge of whether management activities are taking place in a pluvial or a drought could improve the ability of managers to predict the likelihood of success for various objectives. For example, thinning in a pluvial might meet substantive objectives to improve runoff, whereas thinning in a drought might be more significant for reducing risks of catastrophic crown fires or drought mortality.

With the notable exception of a mega-drought, the droughts and pluvials examined in this study were characteristic of climate variability in the region. An examination of hydro-climatic trends in the Salt-Verde watersheds revealed that temperatures are non-stationary, increasing significantly in recent decades, but the same is not true for winter precipitation or resulting stream flows [55]. Rather, winter precipitation has fluctuated between droughts and pluvials in these watersheds in the last century with no evidence of directional change, a pattern that has reoccurred in the southwestern United States for the last 1000 years [50]. These precipitation cycles have been linked to persistent anomalies in sea surface temperatures that vary across inter-annual and decadal time steps [67], [68]. If past patterns of precipitation variability remain stable in the near term, then it is probable that precipitation and flows in the Salt-Verde watersheds will shift into wetter conditions within the timeframe examined in this study [66].

4. *Building Knowledge to Reduce Uncertainties* – Landscape-scale restoration projects like 4FRI present the opportunity to learn about the influence of accelerated thinning on forest water budgets and resilience using modern forestry techniques and under a changing and variable climate. Stakeholders in the 4FRI project are developing a robust adaptive management and monitoring program so that progress towards objectives can be measured and timely adjustments to management made. Additionally, a paired-watershed experiment to evaluate the effects of forest treatments, including prescribed fire maintenance treatments, on watershed properties is planned within the first analysis area of 4FRI. It will monitor runoff, precipitation, evapotranspiration, snow water storage, soil moisture, groundwater recharge, and sediment yield, thereby reducing our knowledge gaps regarding the effects of forest management on the water cycle.

## Conclusions

The widespread pattern of forest drying, evidenced by ecosystem level moisture deficits and larger and more severe wildfires, indicates we are entering a new era of forest management. The 4FRI project is an example of a restoration effort that is addressing this challenge in two ways: establishing objectives to improve resilience and increasing the scale at which management tools are applied. Our study demonstrated that this type of project can increase runoff by approximately 20% compared to unthinned forests, even under simulated drought conditions. If treatments at this scale are completed and repeated over the next several decades, increases in runoff could help offset the current and projected declines in snowpack and stream flow due to warming while improving the resilience of forest stands. As an incidental benefit in an era of dwindling water supplies and projected water shortages, forest thinning could play a role in augmenting river flows on a seasonal basis, improving conditions for water-dependent ecosystems, and benefitting the water supplies of downstream communities. Accelerated forest thinning to reduce water stress and wildfire risk is one of the only management options under our control, and it is probably the most critical to apply over the short term. Because the effects are temporary, it is important to frame accelerated thinning as a reset of ecosystem resilience, not a permanent cure. Rather, accelerated forest thinning creates opportunities to apply other management tools that restore forest resilience at broader scales, whereas management actions at the current pace will likely not be sufficient to address recent and projected changes in forest conditions.

## Supporting Information

File S1 **Methods and results figures and tables in English units.**
(DOCX)

File S2 **Original and revised Baker-Kovner regression models in English units.**
(DOCX)

File S3 **Additional factors that influence runoff.**
(DOCX)

File S4 **Temperature trend analysis.**
(DOCX)
