# Correction: Effects of Climate Variability and Accelerated Forest Thinning on Watershed-Scale Runoff in Southwestern USA Ponderosa Pine Forests

**DOI:** 10.1371/journal.pone.0118044

**Published:** 2015-03-13

**Authors:** The PLOS ONE Staff

There are errors in Fig. 10B and S10 Fig. The symbols are reversed: open circles should be black closed circles, and black closed circles should be open. Please view the corrected Fig. 10 and S10 Fig. here.

**Fig 10:**
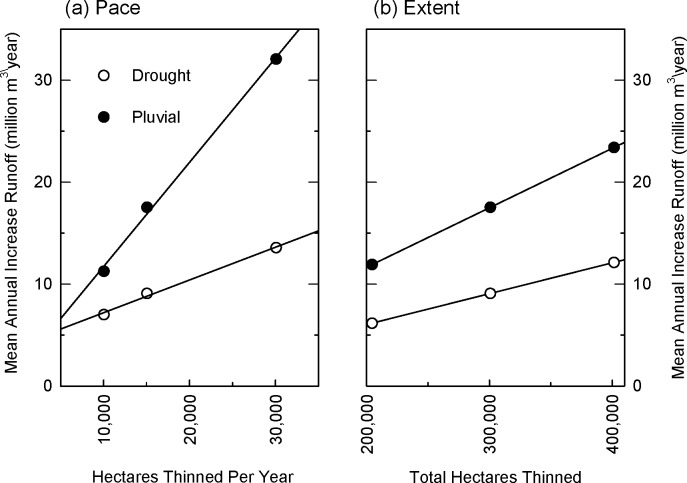
Scale effects of thinning on runoff in Salt-Verde watersheds. Effects of increasing (a) pace and (b) extent of thinning treatments in ponderosa pine forests in Salt-Verde watersheds on increases in mean annual runoff (million m3/year). In (a) total area thinned is held constant at 301,000 ha (743,000 acres) (scenarios: 35mid, 25mid, 15mid) to show influence of increasing the area thinned per year. In (b) duration of thinning treatments is held constant at 25 years (scenarios: 25low, 25mid, 25high) to show influence of increasing the total area thinned across the scenario. In order to illustrate scale effects, only increases in mean annual runoff are shown. Statistics describing annual variability in runoff gains are shown in Table 2 and illustrated graphically for 4FRI scenario in Fig. 8.

## Supporting Information

S10 Fig Scale effects of thinning on runoff in Salt-Verde watersheds. Effects of increasing (a) pace and (b) extent of thinning treatments of ponderosa pine forests in Salt-Verde watersheds on increases in mean annual runoff (acre-feet/year). In (a) total area thinned is held constant at 301,000 ha (743,000 acres) (scenarios: 35mid, 25mid, 15mid) to show influence of increasing the area thinned per year. In (b) duration of thinning treatments is held constant at 25 years (scenarios: 25low, 25mid, 25high) to show influence of increasing the total area thinned across the scenario. In order to illustrate scale effects, only increases in *mean* annual runoff are shown. Statistics describing annual variability in runoff in these scenarios is shown in Table 2 and illustrated graphically for 4FRI scenario in S8 Fig.
(TIFF)
