# Supplementary material for: Effects of Climate Variability and Accelerated Forest Thinning on Watershed-Scale Runoff in Southwestern USA Ponderosa Pine Forests
Source: PLoS One. 2014 Oct 22;9(10):e111092. doi: 10.1371/journal.pone.0111092 (PMC4206497; doi:10.1371/journal.pone.0111092)
Supplement: File S1 — Methods and results figures and tables in English units. (DOCX) [file pone.0111092.s001.docx]

**File S1: Methods and results figures and tables in English Units**

**Figure S1. Map of Study Area.** Map showing ponderosa pine forests in Salt-Verde watersheds in central Arizona, including those forests that are slated for mechanical thinning within the 4FRI project. Runoff from snowmelt in these forests is primary source of flow to Salt-Verde rivers which in turn are major sources of water for communities in the Phoenix Metro Area. Study used runoff model developed from experimental studies conducted in Beaver Creek watershed (Brown et al. 1974). *Inset*: Location of study area in Arizona.


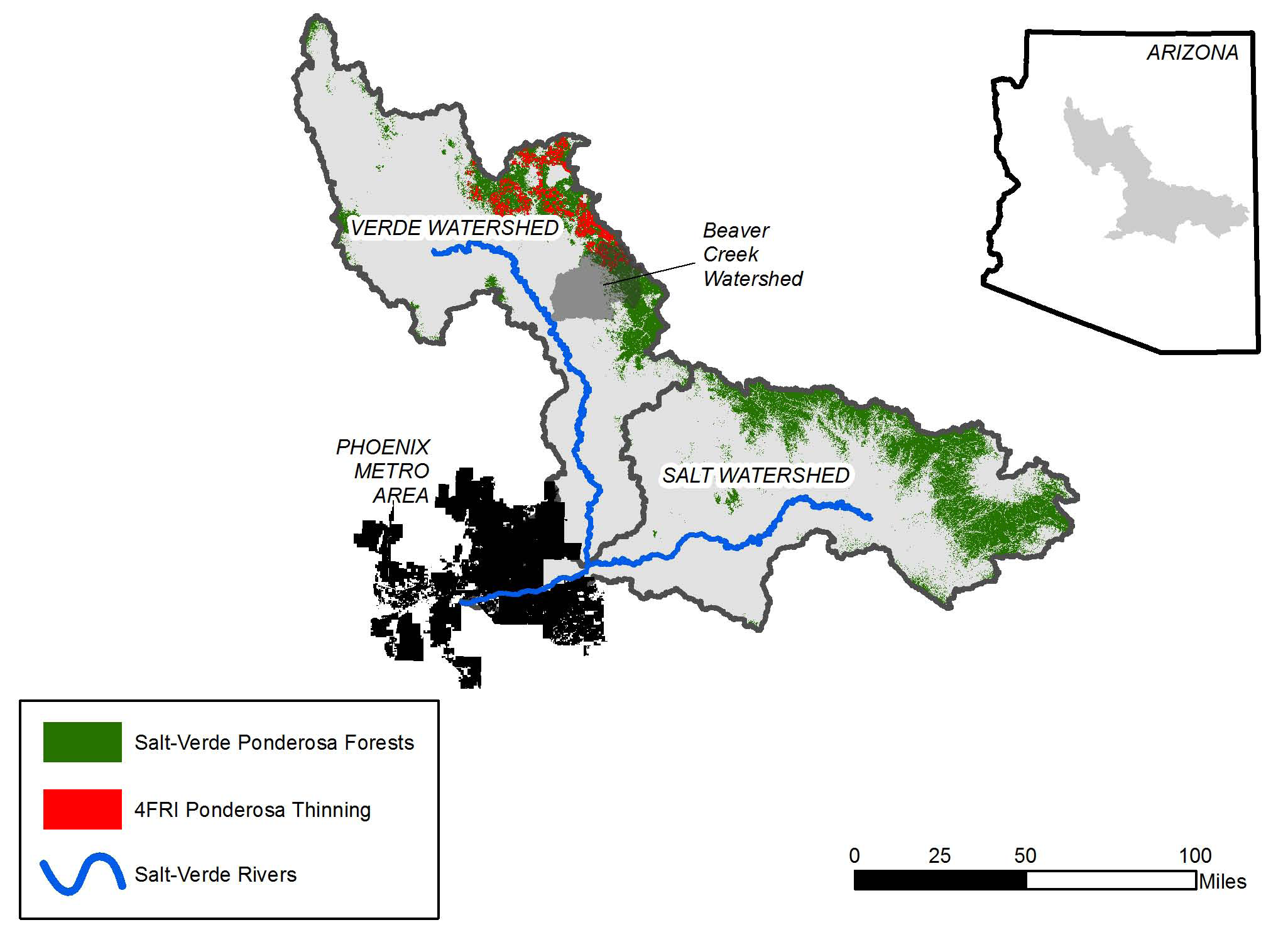


**Figure S2. Comparison of models to observed runoff.** Fit of (a) original (Brown et al. 1974) and (b) modified Baker-Kovner regression model output to increases in runoff associated with forests treatments in central Arizona, from Beaver Creek (Baker 1986, Neary 2011) and Castle Creek (Rich 1972) watersheds.


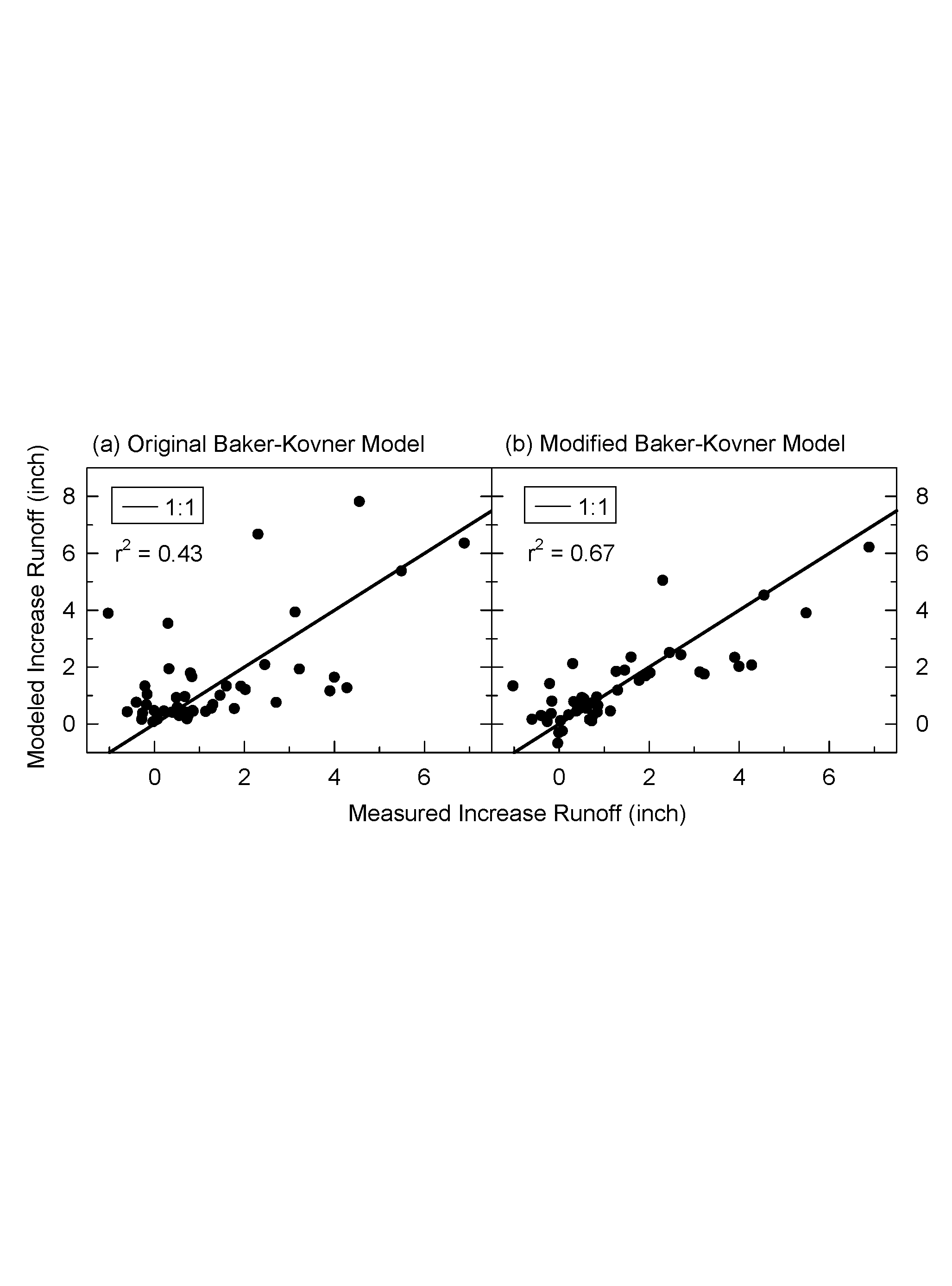


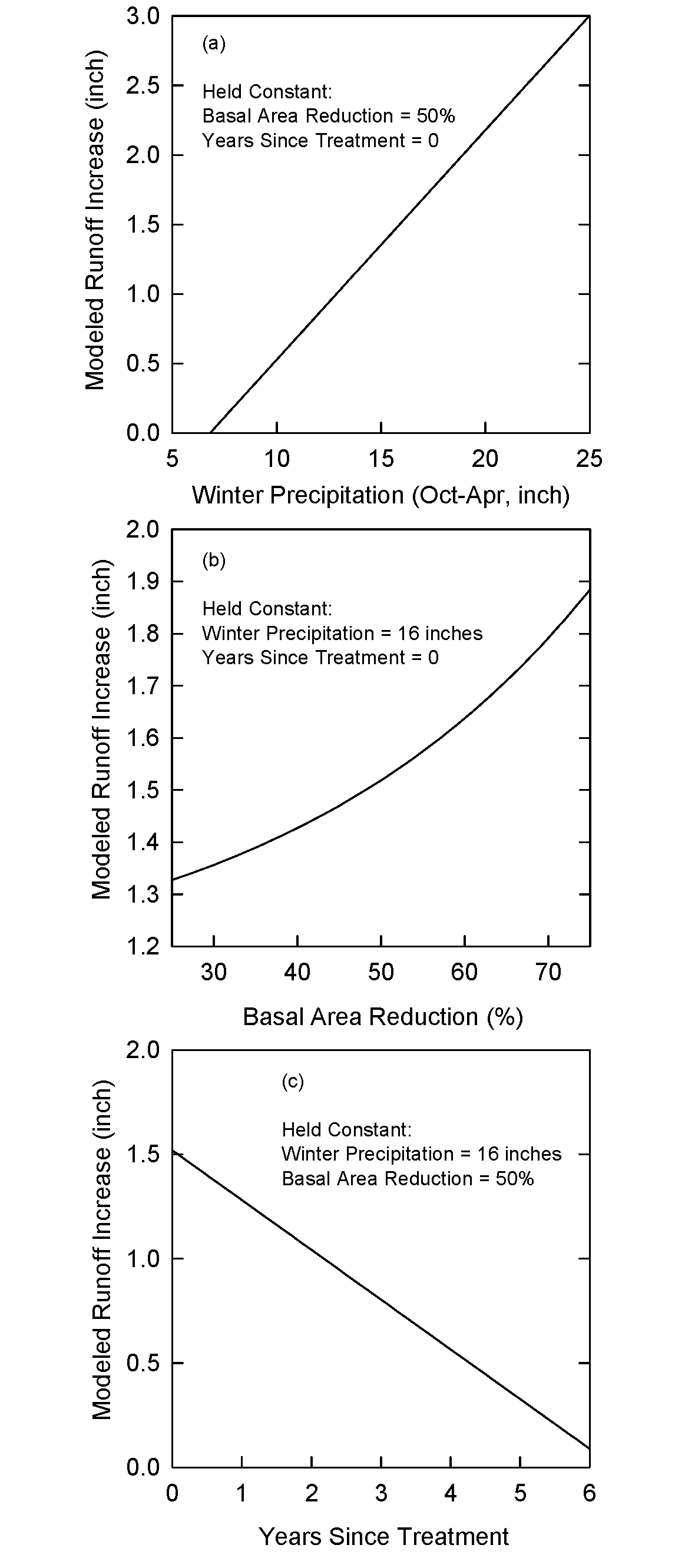
**Figure S3. Influence of independent variables on runoff model.** Relationships between model output to values for independent variables, including (a) winter precipitation, (b) percent basal area reduction, and (c) years since treatment. In all cases, other independent variables are held constant in order to view relationship of independent variable plotted on X-axis to model output.

**Figure S4. Variability in winter precipitation in ponderosa pine forests.** Estimates of historical winter precipitation from 1900-2012 in ponderosa pine forests within Verde watershed from PRISM model (Daly et al. 2008). Shaded areas are examples of 15-year droughts and pluvials that were used in study scenarios; horizontal red lines represent mean winter precipitation within these shaded areas. *Inset:* Comparison of measured winter precipitation observed during the historical Beaver Creek watershed experiments (Brown et al. 1974) from 1958-1982 versus modeled winter precipitation data shown in main figure.


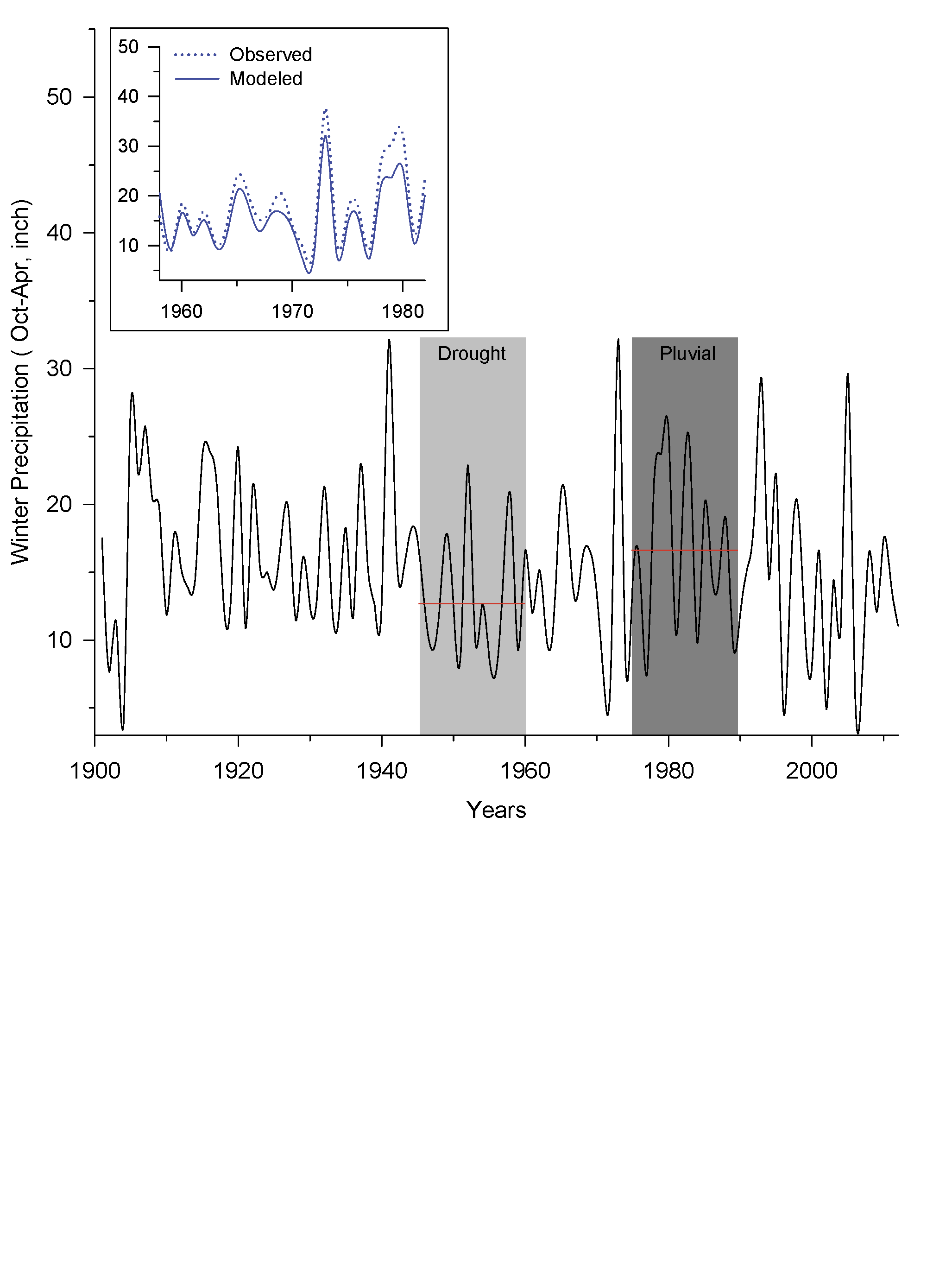


**Figure S5. Ponderosa pine basal area reductions in 4FRI project.** Histograms showing (top) pre-thinning and (bottom) desired post-thinning basal areas (in ft^2^/acre) of ponderosa pine stands in the first analysis area of the 4FRI project (excluding stands where basal area reduction <= 30%).


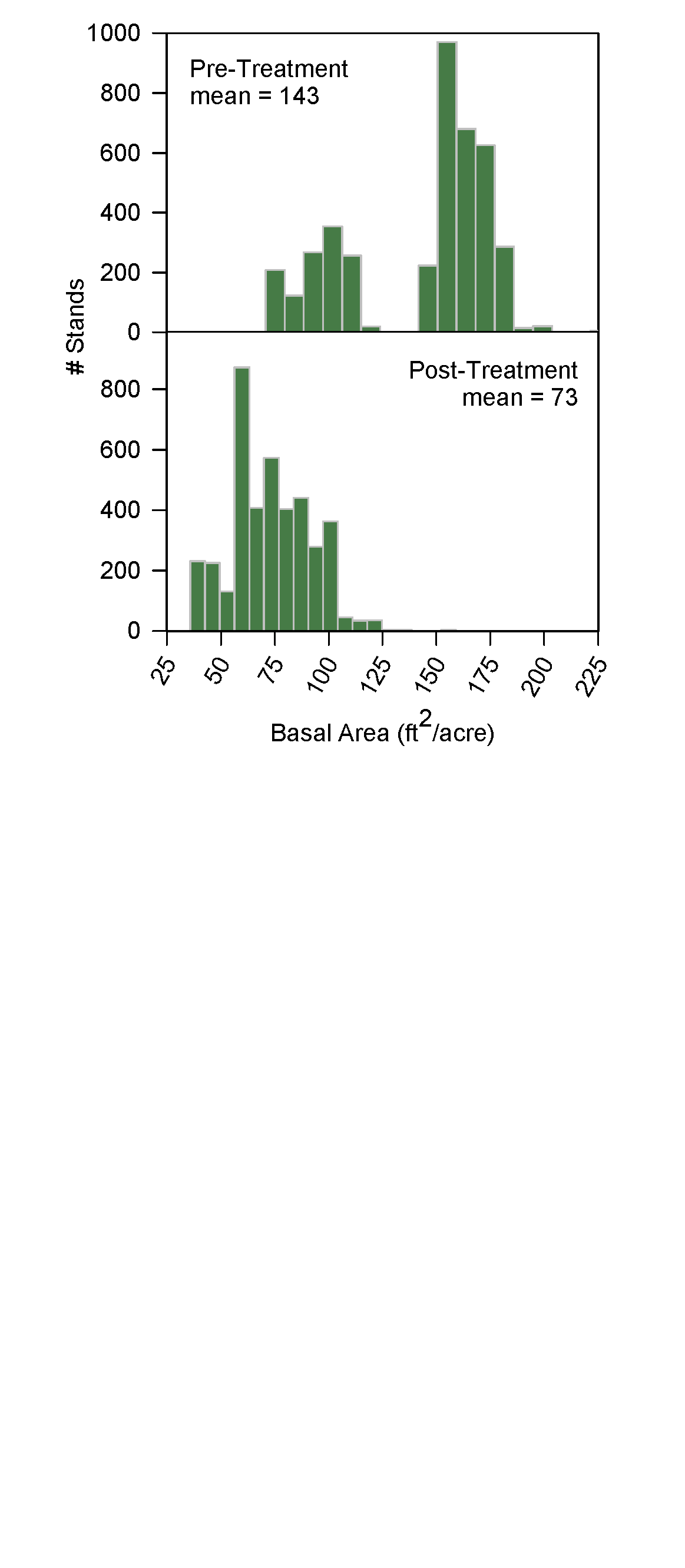


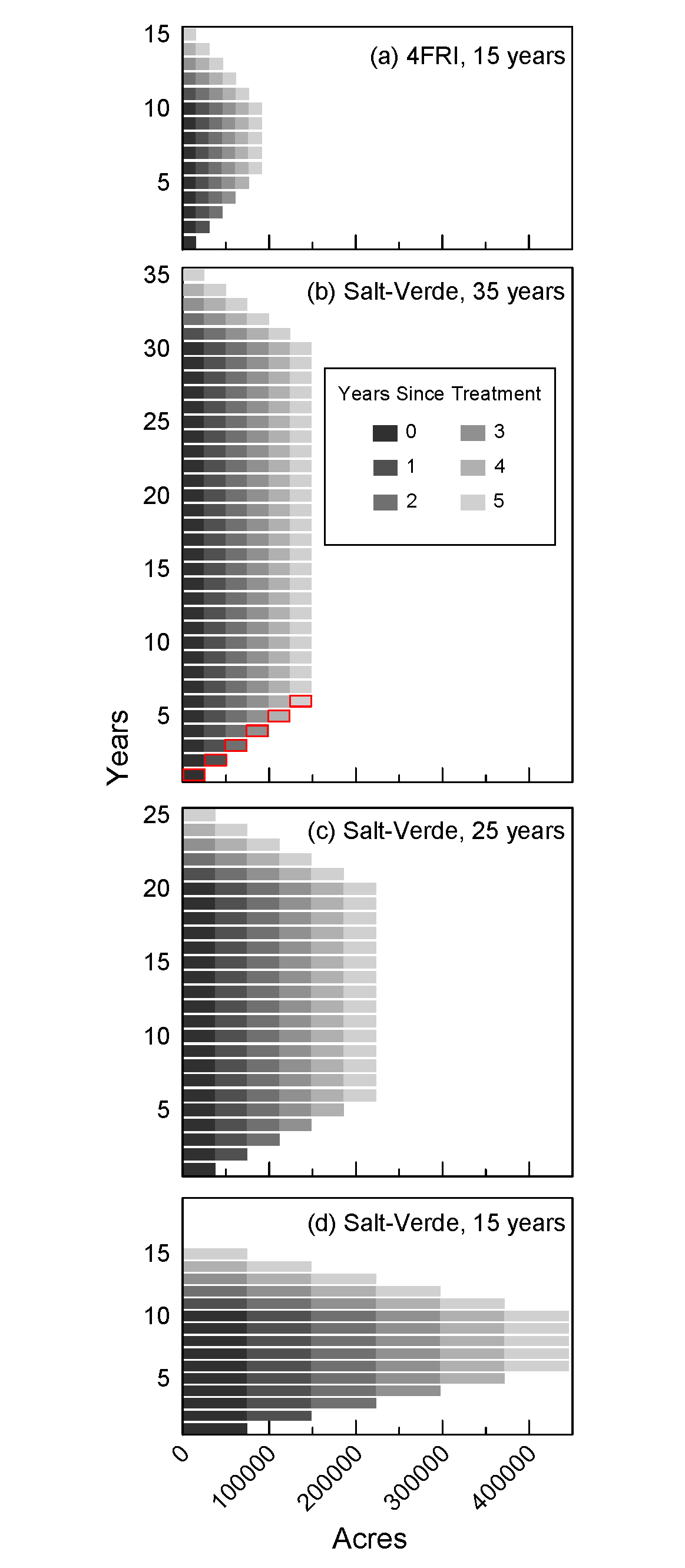
**Figure S6. Forest treatment schedules for study scenarios.** Graphical depiction of mechanical thinning treatment schedules for (a) 15-year 4FRI scenarios and (b-d) 35-year, 25-year, and 15-year Salt-Verde moderate thinning scenarios (total thinned area was 301,000 ha or 743,000 acres). Scenarios assumed consecutive treatments for 10-, 20-, and 30-year treatment periods shown as black bars in the bottom left portion of each of the figures. Bars outlined in red show the contribution of one cohort of stands through six years in the scenario.

**Figure S7. Increases in mean annual runoff from thinning in 4FRI project.** Results from 26 scenarios with varying levels of winter precipitation showing increases in mean annual runoff associated with mechanical thinning of ponderosa pine forests in the first analysis area of the 4FRI project. In order to compare scenarios, only increases in *mean* annual runoff are shown. Annual variability in runoff for two of these scenarios is shown in Figure S8.


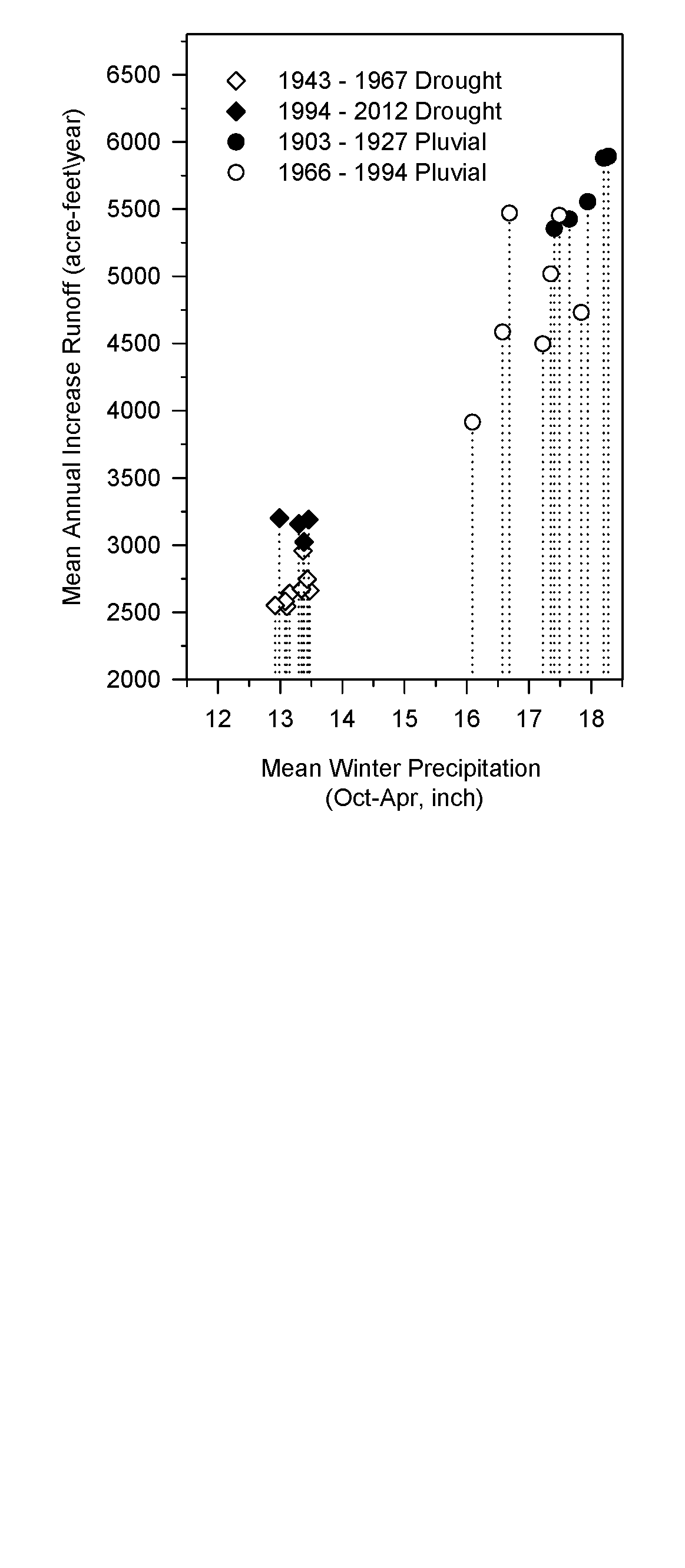


**Figure S8. Year to year variability in runoff increases from thinning in 4FRI project.** Modeled increases in annual runoff associated with mechanical thinning of ponderosa pine forests in the first analysis area of the 4FRI project during the (a) drought and (b) pluvial that produced lowest and highest runoff levels respectively. Top panes show increases in runoff in acre-feet. Solid black lines are output values from regression model; dotted lines and blue areas represent 90% confidence intervals. Bottom panes show corresponding winter precipitation values (Oct-Apr, inches) used as independent variable to calculate runoff.


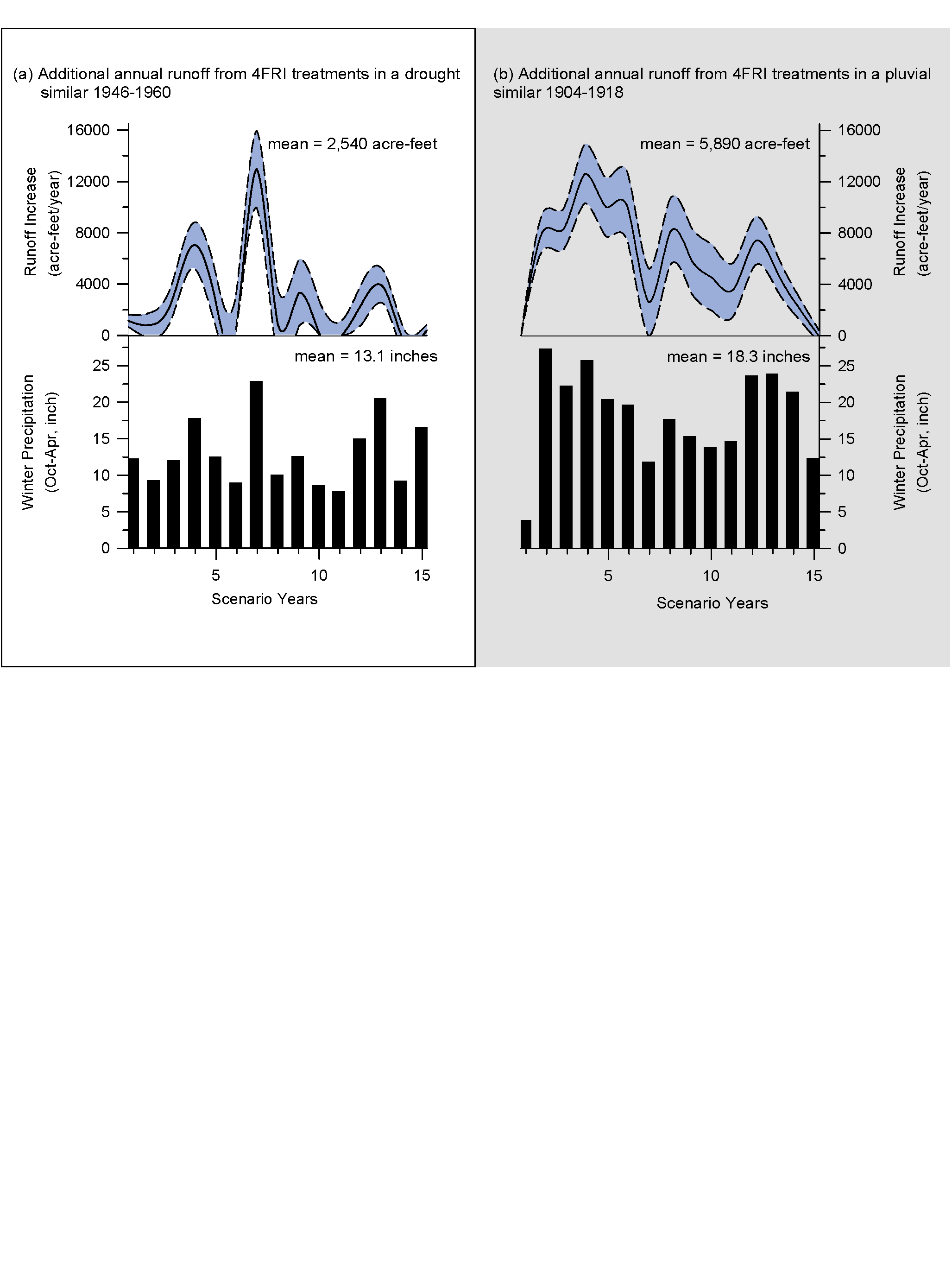


**Figure S9. Cumulative runoff increases from thinning in 4FRI project.** Estimates of cumulative increases in runoff (acre-feet) from planned mechanical thinning of ponderosa pine forests in the first analysis area of the 4FRI project under (a) drought and (b) pluvial conditions. Solid red lines are estimates of cumulative runoff under current forest conditions using original Baker-Kovner regression model (Brown et al. 1974). Dotted red lines represent increases in cumulative runoff associated with 4FRI treatments using modified Baker-Kovner regression model. Difference between these two values, shown with blue shading, is additional runoff from forest thinning treatments. Estimated increases in runoff ceased after 15 years.


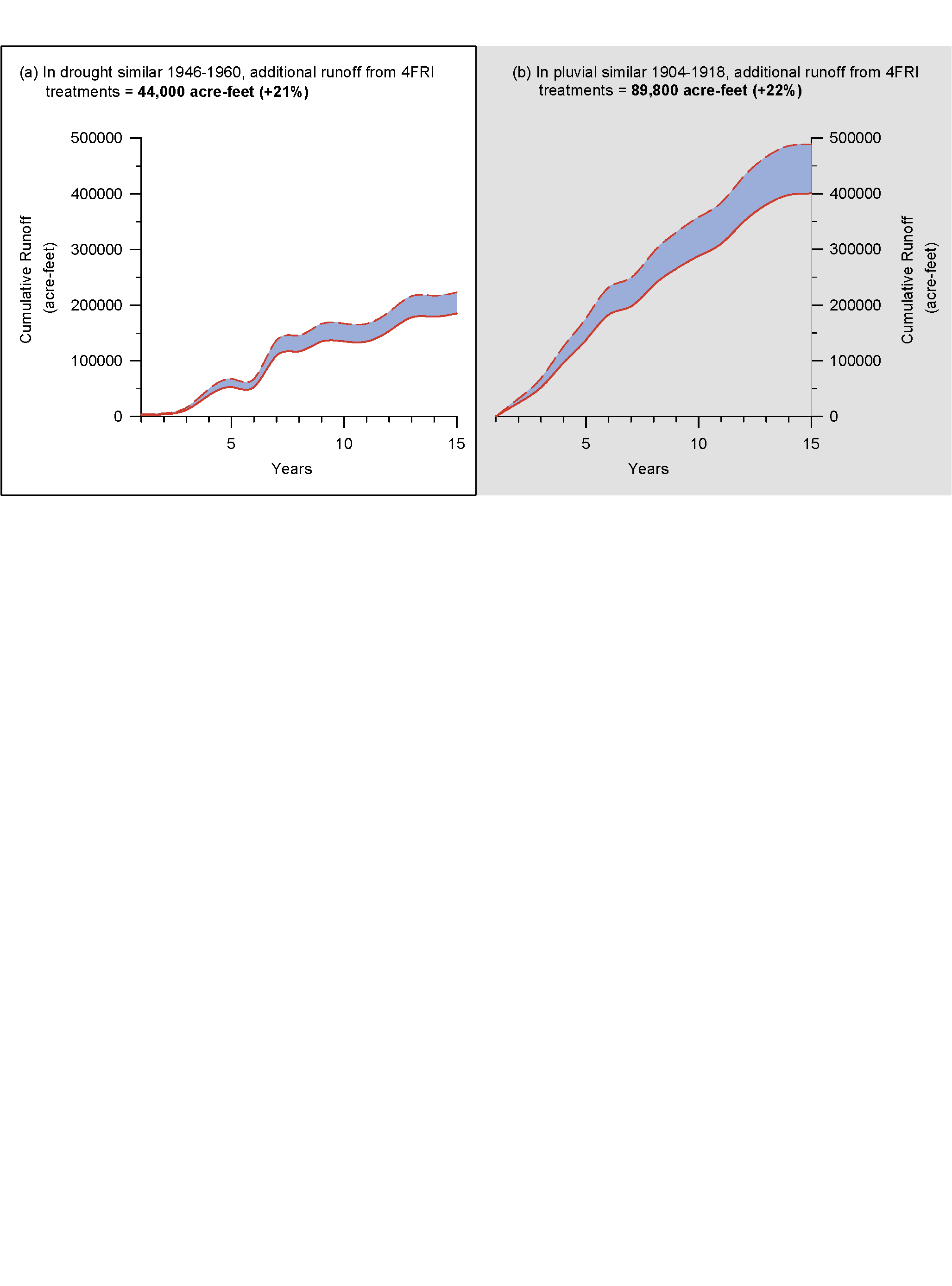


**Figure S10. Scale effects of thinning on runoff in Salt-Verde watersheds.** Effects of increasing (a) pace and (b) extent of thinning treatments of ponderosa pine forests in Salt-Verde watersheds on increases in mean annual runoff (acre-feet/year). In (a) total area thinned is held constant at 301,000 ha (743,000 acres) (scenarios: 35mid, 25mid, 15mid) to show influence of increasing the area thinned per year. In (b) duration of thinning treatments is held constant at 25 years (scenarios: 25low, 25mid, 25high) to show influence of increasing the total area thinned across the scenario. In order to illustrate scale effects, only increases in *mean* annual runoff are shown. Statistics describing annual variability in runoff in these scenarios is shown in Table 2 and illustrated graphically for 4FRI scenario in Figure S8.


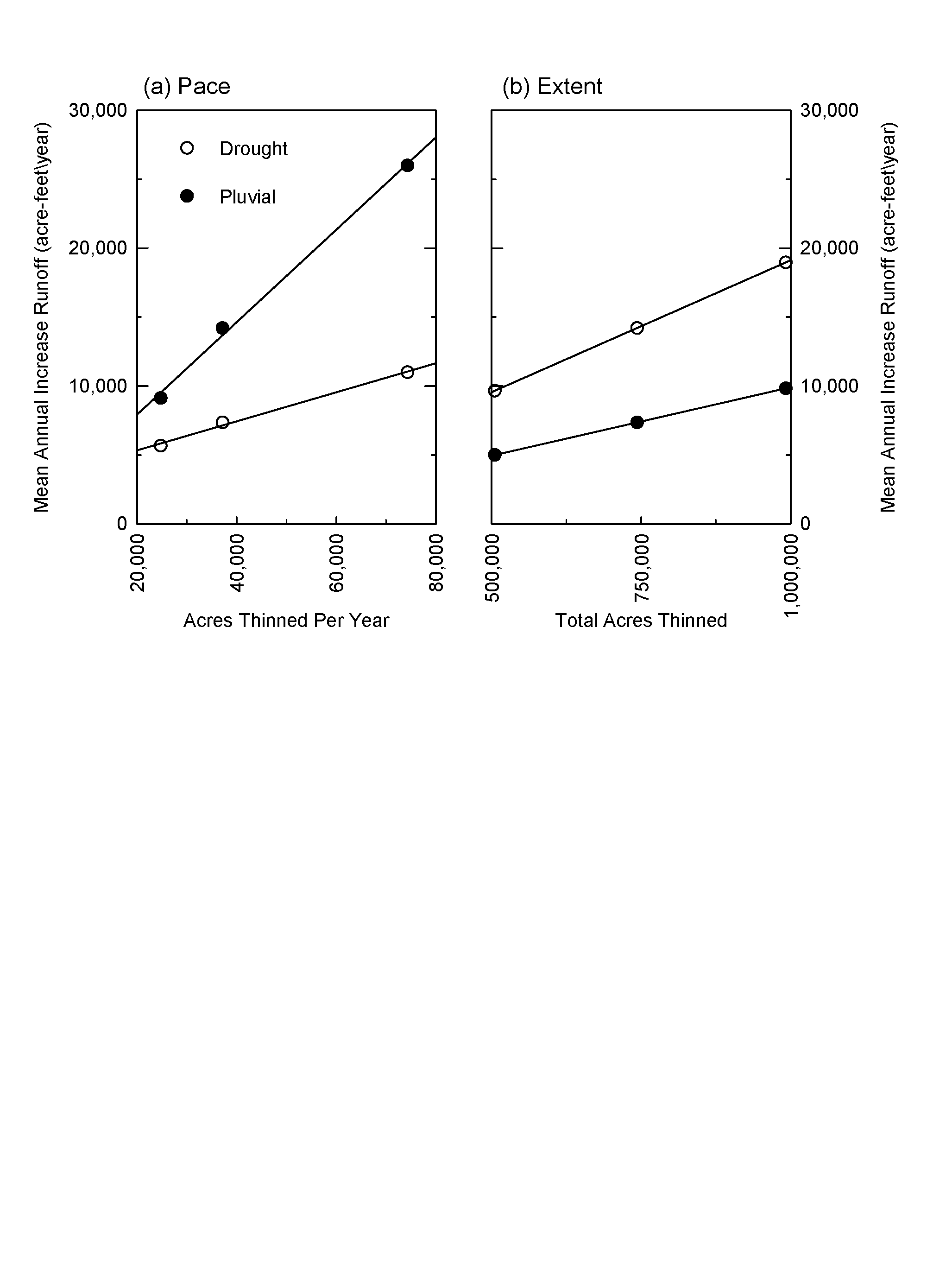


**Table S1. Estimate of acres of ponderosa pine forests available to mechanical thinning in Salt-Verde watersheds.** Summary of acres and percentages of ponderosa pine forests within Salt-Verde watersheds in relation to potential for thinning treatments, including total acres, those acres typically excluded from mechanical thinning, and the remaining acres available for mechanical thinning. Estimates rounded off to three significant digits.

| **Category** | | **Acres** | **%** | ***Sources*** |
| --- | --- | --- | --- | --- |
| *Salt-Verde Watersheds Ponderosa Pine Forests* | | *1,684,000* | *-* | USGS-GAP 2010 |
| *Exclusions* | |  |  |  |
|  | *Steep Slopes* greater than or equal to 40% | *168,000* | 10% | Gesch et al. 2002 |
|  | *Hi-Severity Burn Patches* within Wallow and Rodeo-Chedeski Fires | *108,000* | 6% | Eidenshink et al. 2007 |
|  | *Specially Designated Areas* including  Wilderness Areas, Research Natural Areas, State Wildlife Management Areas ^a^ | *53,800* | 3% | CBI 2010; USDA-FS 2013 |
|  | *Streamside Management Zones* within 100 feet perennial reaches in national forests and 200 feet in tribal areas | *19,900* | 1% | Turner and List 2007 |
|  | *Mexican Spotted Owl Protected Activity Centers* where owls have been found to be nesting^b^ | *142,000* | 8% | Hampton et al. 2011 |
|  | *Erodible Soils* where thinning is unsuitable due to erosion risk or rocky conditions ^c^ | *230,000* | 14% | Hampton et al. 2011; USDA-FS 2013 |
|  | *Completed Treatments* that have been thinned in last 10 years ^d^ | *87,100* | 5% | USDA-FS 2013 |
| *Sub-Total Exclusions (accounting for overlap between layers)* | | *692,000* | *41%* |  |
| *Sub-Total Forest Available Mechanical Thinning* | | *992,000* | *59%* |  |

^a^ Estimate derived from Southwestern Region Wilderness Status layer and Forest Level Special Interest Management Areas layers (USDA-FS 2013).

^b^ Data available for Coconino, Apache-Sitgreaves, Kaibab, and Tonto National Forests only. Added qualitative estimate for this exclusion factor for tribal lands and Prescott National Forest assuming land excluded in these land units for this factor would be proportional to lands excluded for this factor on the four national forests above.

^c^ List of excluded soils for Coconino, Apache-Sitgreaves, Kaibab, and Tonto National Forests based on Hampton et al. (2011). This list of excluded soils was augmented based on consultation with southwestern region USDA-FS soil scientists. Geospatial data for these soils derived from Forest Level Terrestrial Ecological Units layers (USDA-FS 2013). Added qualitative estimate for this exclusion factor for tribal lands and Prescott National Forest where geospatial data was unavailable, assuming land excluded in these land units for this factor would be proportional to lands excluded for this factor on the four national forests above.

^d^ Geospatial data for this factor derived from Forest Level Activities Layer joined to FACTs table for Coconino, Apache-Sitgreaves, Kaibab, Tonto, and Prescott National Forests (USDA-FS 2013). Added qualitative estimate for this exclusion factor for tribal lands based on consultation with Bureau of Indian Affairs forestry personnel.

**Table S2. Annual runoff increases from thinning in Salt-Verde watersheds.** Estimates of additional runoff associated with thinning of ponderosa pine forests across Salt-Verde watersheds accounting for variation in treatment periods, total forest extent treated, and drought and pluvial periods of winter precipitation (Oct-Apr). Estimates rounded off to three significant digits.

| **Forest Management^a^** | | | | **Winter Precipitation Regime** | | | **Runoff: Annual Increase** | | | **Runoff: Cumulative Increase** | |
| --- | --- | --- | --- | --- | --- | --- | --- | --- | --- | --- | --- |
| *Scenario Name* | *Total Acres* | *Acres per Year* | *Effective Acres per Year* | *Type* | *Similar to 20th Century Period* | *Mean (In/Yr)* | *Mean (acre-feet)* | *Median (acre-feet)* | *Maximum (acre-feet)* | *Total (acre-feet)* | *%* |
| 35-low | 505,000 | 16,800 | 86,600 | Drought | 1942-1976 | 13.4 | 3,860 | 2,420 | 14,200 | 135,000 | 23% |
|  |  |  |  | Pluvial | 1965-1999 | 15.6 | 6,200 | 4,740 | 20,800 | 217,000 | 26% |
| 35-mid | 743,000 | 24,800 | 127,000 | Drought | 1942-1976 | 13.4 | 5,680 | 3,560 | 20,900 | 199,000 | 23% |
|  |  |  |  | Pluvial | 1965-1999 | 15.6 | 9,130 | 6,980 | 30,600 | 319,000 | 26% |
| 35-high | 992,000 | 33,100 | 170,000 | Drought | 1942-1976 | 13.4 | 7,580 | 4,750 | 27,900 | 265,000 | 23% |
|  |  |  |  | Pluvial | 1965-1999 | 15.6 | 12,200 | 9,320 | 40,900 | 426,000 | 26% |
| 25-low | 505,000 | 25,300 | 121,000 | Drought | 1942-1966 | 13.3 | 5,010 | 3,290 | 21,300 | 125,000 | 20% |
|  |  |  |  | Pluvial | 1975-1999 | 16.2 | 9,660 | 7,820 | 27,200 | 242,000 | 22% |
| 25-mid | 743,000 | 37,200 | 178,000 | Drought | 1942-1966 | 13.3 | 7,370 | 4,840 | 31,400 | 184,000 | 20% |
|  |  |  |  | Pluvial | 1975-1999 | 16.2 | 14,200 | 11,500 | 40,000 | 355,000 | 22% |
| 25-high | 992,000 | 49,600 | 238,000 | Drought | 1942-1966 | 13.3 | 9,830 | 6,470 | 41,900 | 246,000 | 20% |
|  |  |  |  | Pluvial | 1975-1999 | 16.2 | 19,000 | 15,400 | 53,500 | 474,000 | 22% |
| 15-low | 505,000 | 50,500 | 202,000 | Drought | 1945-1959 | 12.4 | 7,480 | 4,820 | 42,400 | 112,000 | 20% |
|  |  |  |  | Pluvial | 1975-1989 | 16.6 | 17,700 | 6,740 | 48,700 | 265,000 | 22% |
| 15-mid | 743,000 | 74,300 | 297,000 | Drought | 1945-1959 | 12.4 | 11,000 | 7,090 | 62,400 | 165,000 | 20% |
|  |  |  |  | Pluvial | 1975-1989 | 16.6 | 26,000 | 9,910 | 71,700 | 390,000 | 22% |
| 15-high | 992,000 | 99,200 | 397,000 | Drought | 1945-1959 | 12.4 | 14,700 | 9,470 | 83,300 | 220,000 | 20% |
|  |  |  |  | Pluvial | 1975-1989 | 16.6 | 34,700 | 13,200 | 95,700 | 521,000 | 22% |

^a^ ‘Scenario Name’ indicates number of years simulated in each scenario (15, 25, 35 years) and extent of forest thinned (low = 30% of forest thinned; mid = 45%; high = 60%). ‘Total Acres’ are total number of acres thinned in each scenario. ‘Acres per Year’ are number of acres thinned every year for 10 consecutive years (15-year scenarios), 20 consecutive years (25-year scenarios), and 30 consecutive years (35-year scenarios). ‘Effective Acres per Year’ are the average number of thinned acres across all years of the scenario that contribute to additional runoff (see figure S6).
