# Supplementary material for: Effects of Climate Variability and Accelerated Forest Thinning on Watershed-Scale Runoff in Southwestern USA Ponderosa Pine Forests
Source: PLoS One. 2014 Oct 22;9(10):e111092. doi: 10.1371/journal.pone.0111092 (PMC4206497; doi:10.1371/journal.pone.0111092)
Supplement: File S2 — Original and revised Baker-Kovner regression models in English units. (DOCX) [file pone.0111092.s002.docx]

File S2. Original and revised Baker-Kovner regression models in English Units

The original Baker-Kovner regression model (Baker et al. 1974) estimated total runoff at the subwatershed outlet. In English Units, it can be expressed as:

R_watershed_ = -5.72 + 0.83*P + 0.42*r – 0.24*r*P^0.92^ – 0.007*P^2^*(1 – e^-BA/45^)^3^

r^2^ = 0.69 , where

R_watershed_ = Annual Runoff in watershed in inches

P = Total Winter Precipitation (Oct-Apr) in inches

r = Insolation as a decimal fraction

BA = Basal Area in ft^2^/acre

The revised Baker-Kovner regression model (this study) estimated additional runoff from forest thinning at the subwatershed outlet. In English Units, it can be expressed as:

R_treatment_ = -1.121 + 0.148*P – 0.015*P*Y – 0.092*P*[exp(– BA_1_/45) – exp(– BA_2_/45)]

r^2^ = 0.67, where

R_treatment_ = Increase in Annual Runoff attributed to Forest Thinning in inches

P = Total Winter Precipitation (Oct-Apr) in inches

Y = Years since Treatment

BA_1_ = Basal Area before Treatment in ft^2^/acre

BA_2_ = Basal Area after Treatment in ft^2^/acre
