# Supplementary material for: Effects of Climate Variability and Accelerated Forest Thinning on Watershed-Scale Runoff in Southwestern USA Ponderosa Pine Forests
Source: PLoS One. 2014 Oct 22;9(10):e111092. doi: 10.1371/journal.pone.0111092 (PMC4206497; doi:10.1371/journal.pone.0111092)
Supplement: File S3 — Additional factors that influence runoff. (DOCX) [file pone.0111092.s003.docx]

**File S3. Additional factors that influence runoff.** List of additional factors that influence runoff patterns in ponderosa pine ecosystems, including evidence and uncertainties associated with the current and projected future effects of factors on runoff. Direction of influence includes: ‘+’ factor likely to increase range of runoff reported in this study; ‘-‘ factor likely to decrease range of runoff reported in this study; ‘?’ effects of factor have not measured, uncertain, or have confounding effects on runoff.

| **Factor** | **Description** | **Direction of Influence on Runoff** | **References** |
| --- | --- | --- | --- |
| ***CLIMATE CHANGE*** | | | |
| Changes in winter precipitation | Global Circulation models (GCMs) project mean precipitation will decline between 0-10% by the end of the century. Not all GCMs agree on direction or magnitude. Single-digit declines may not be detectable from natural variability. GCMs project increase in frequency and intensity of extreme precipitation events and subsequent flooding. This will likely change daily hydrograph and peak flows, but it is not clear how this factor will influence magnitude and direction of seasonal or annual runoff given contributions and influence of other climatic drivers including warming. | ? | Dominguez et al. 2012; Garfin et al. 2013 |
| Warming-induced decline in snowpack levels | Recent declines in snowpack levels in West and Arizona have been observed. Portion of decline is due to recent drought, whereas additional portion may be due to elevated temperatures. GCMs project warming-induced reduction in winter mountain snowpack will continue. | - | Mote 2006; Ffolliott and Gottfried 2010; Garfin et al. 2013 |
| Warming-induced increase in severe wildfires | In recent decades, size and severity of wildfires have increases in the West. A portion of this increase in wildfire activity is likely due to recent warming although other factors such as previous land use contribute as well. Trend of increased severity and size of wildfires are projected to continue. Studies show that wildfires can result in significant increases in runoff quantity (also sedimentation and erosion), especially peak flows after large storms. Effects are more pronounced in high severity burn areas. Peak flow levels can return to pre-fire levels several years after fire (~5 years) as vegetation recovers. State change following wildfires from forests to grassland could permanently change hydrologic functioning including runoff patterns. | + | Campbell et al. 1977; Helvey 1980; Benavides-Solorio et al. 2001; Westerling et al. 2006; Ffolliott et al. 2011; Williams et al. 2013 |
| Warming-induced Drought Mortality | Some evidence that warming is already contributing to an increase in the size and severity of tree mortality in forests and woodlands under drought conditions. Drought mortality trends are projected to continue and worsen. We could find no empirical studies that measured effects of ponderosa pine mortality on runoff. | ? | Breshears et al. 2005; Ganey and Vojta 2011; Williams et al. 2013 |
| ***CLIMATE VARIABILITY*** | | | |
| Droughts and Pluvials | Scenarios of additional runoff associated with thinning in this study used model of 20th century winter precipitation. Examination of paleoclimate record suggests that 20th century record may capture high range variability associated with pluvials but not lower range associated with droughts. Early 20th century pluvial in this study may have been one the most extreme pluvial period that has occurred in last 12 centuries. However, there is evidence of more extreme droughts than the two occurrences of drought in 20th century. | - | Woodhouse and Overpeck 1998; Stahle et al. 2000; Ni et al. 2002; Woodhouse et al. 2005; Cook et al. 2007 |
| Years with above average precipitation | This study used PRISM model of 20th century precipitation. Estimates of winter precipitation in PRISM model were on average 1 inch less than direct measurements of precipitation taken during Beaver Creek watershed experiments. The model consistently under-predicted levels of winter precipitation in wet years. | + | Brown et al. 1974; Daly et al. 2008 |
| ***HYDROGEOLOGY*** | | | |
| Transmission Losses | This study estimated additional "in-situ" runoff associated with thinning at subwatershed outlets. One modeling study suggested that less than one-half of runoff gains from thinning would reach downstream users. More precise data and knowledge regarding losses to groundwater recharge, riparian area transpiration and evapotranspiration are lacking. | - | Brown and Fogel 1987; Ffolliott 2007 |
| Soil Infiltration & Recharge Rates | This study adapted a regression model developed for runoff from ponderosa pine forest treatments on volcanic (basalt-derived) soils. Based on a four-year study of untreated ponderosa pine watersheds on sedimentary soils, annual water yield was about 25% of water yield from volcanic soils. | ? | Brown et al. 1974; Ffolliott and Baker 1977 |
| ***FOREST MANAGEMENT*** | | | |
| Thinning of Mixed Conifer Forests | On a per-acre basis, increases in runoff were higher and effects lasted longer when mixed conifer forests were thinned as compared to thinning of ponderosa pine forests. These forests generally occur at higher elevations with higher mean annual precipitation. The first analysis area of 4FRI and subsequent phases will include thinning of mixed conifer forests thought these forests comprise a much smaller portion of Salt-Verde watersheds. | + | Rich and Gottfried 1976; Gottfried 1991; Baker 1999 |
| Maintenance Treatments | Additional runoff with forest thinning declined with years-since-treatment in historic watershed experiments. Researchers hypothesized that declines were due to regrowth of understory vegetation. One study found understory evapotranspiration was found to be higher than overstory evapotranspiration in a thinned stand. This suggests that maintenance treatments, including prescribed fires, to remove this understory could have the effect of maintaining increased runoff levels. | + | Baker 2003; Simonin et al. 2007 |
| Thinning Prescriptions | Influence of size and configuration of open patches affects snowpack accumulation and ablation. Even though range of basal area reductions from Beaver Creek experiments were similar to this study, configuration of thinning treatments and creation of open patches were substantially different between these historic experiments (including strip-thinning) and modern thinning prescriptions. The effects of these differences on runoff are unknown. Studies have shown that forests with intermediate canopy cover have the highest snowpack levels because they have a balance between canopy interception of snowfall, which is highest in a closed canopy, and exposure of ground snowpack to radiation, which is highest in an open canopy. | ? | Musselman et al. 2008; Veatch et al. 2009; Biderman et al. 2014 |

**Additional References**

Baker M (1999) History of Watershed Research in the Central Arizona Highlands. General Technical Report RMRS-GTR-29. Fort Collins, CO: U.S. Department of Agriculture, Forest Service, Rocky Mountain Research Station. 56 p.

Benavides-Solorio J, MacDonald LH (2001) Post-ﬁre runoff and erosion from simulated rainfall on small plots, Colorado Front Range. Hydrol. Process. 15: 2931–2952.

Biederman JA, Brooks PD, Harpold AA, Gochis DJ, Gutmann E, et al. (2014) Multiscale observations of snow accumulation and peak snowpack following widespread, insect-induced lodgepole pine mortality. Ecohydrology 7(1): 150–62. doi:10.1002/eco.1342.

Dominguez F, Rivera E, Lettenmaier DP, Castro CL (2012) Changes in winter precipitation extremes for the western United States under a warmer climate as simulated by regional climate models. Geophyscial Research Letters 39(5): 1-7.

Ffolliott PF, Gottfried GJ (2010) Magnitudes and timing of seasonal snowpack water equivalents in Arizona: A preliminary study of the possible effects of recent climate change. Journal of the Arizona-Nevada Academy of Science 42(1): 1-4.

Ffolliott PF, Stropki CL, Chen H, Neary DG (2011) The 2002 Rodeo-Chediski wildfire’s impacts on southwestern ponderosa pine ecosystems, hydrology, and fuels. Research Paper RMRS-RP-85. Fort Collins, CO: U.S. Department of Agriculture, Forest Service, Rocky Mountain Research Station. 36 p.

Ffolliott PF (2007) Extrapolation of water-yield improvement studies on upland watersheds to larger river basins. Hydrology and Water Resources in Arizona and the Southwest: Proceedings of the 2007 meetings of the Hydrology Section, Arizona-Nevada Academy of Science, March 31, 2007. Available: <http://arizona.openrepository.com/arizona/bitstream/10150/296680/1/hwr_37-009-014.pdf>. Accessed 9 April 2013.

Ganey JL, Vojta SC (2011) Tree mortality in drought-stressed mixed-conifer and ponderosa pine forests, Arizona, USA. Forest Ecology and Management 261: 162-168.

Gottfried GJ (1991) Moderate timber harvesting increases water yields from an Arizona mixed conifer watershed. JAWRA Journal of the American Water Resources Association 27 (3): 537–46. doi:10.1111/j.1752-1688.1991.tb01454.x.

Helvey, JD (1980) Effects of a north central Washington wildfire on runoff and sediment production. Journal of the American Water Resources Association. 16(4): 627-634.

Musselman KN, Molotch NP, Brooks PD (2008) Effects of vegetation on snow accumulation and ablation in a mid-latitude sub-alpine forest. Hydrological Processes 22(15): 2767–76.

Simonin K, Kolb TE, Montes-Helu M, Koch GW (2007) The influence of thinning on components of stand water balance in a ponderosa pine forest stand during and after extreme drought. Agricultural and Forest Meteorology 143(3–4): 266–76.

Veatch W, Brooks PD, Gustafson JR, Molotch NP (2009) Quantifying the effects of forest canopy cover on net snow accumulation at a continental, mid-latitude site. Ecohydrology 2: 115-128.
