# Supplementary material for: Effects of Climate Variability and Accelerated Forest Thinning on Watershed-Scale Runoff in Southwestern USA Ponderosa Pine Forests
Source: PLoS One. 2014 Oct 22;9(10):e111092. doi: 10.1371/journal.pone.0111092 (PMC4206497; doi:10.1371/journal.pone.0111092)
Supplement: File S4 — Temperature trend analysis. (DOCX) [file pone.0111092.s004.docx]

**File S4. Temperature trend analysis.**

We found that this distribution of temperatures extracted from the 1900-2012 PRISM model was non-normal for some months so we used non-parametric statistics to evaluate changes in temperatures. To test the accuracy of the modeled temperature data, we ran a Spearman’s rank order correlation comparing monthly mean values from the PRISM model to measurements of mean temperatures recorded from 1958-1982 (Neary 2011). For all seven winter and spring months tested (Oct-Apr), we found a strong association between modeled and observed data (Rho values ranged from 0.935 to 0.971, all p-values < 0.001). We also plotted observed versus modeled data on 1:1 graphs and found no discernable patterns of dispersion or bias. To test for differences in temperatures, we used the Mann Whitney U test to evaluate whether monthly mean temperatures in the last 25 years (1988-2012) in PRISM model differed from temperatures recorded during the 25 years where we have Beaver Creek observation data (1958-1982).
