# Supplementary figures and images for: Correction: Effects of Climate Variability and Accelerated Forest Thinning on Watershed-Scale Runoff in Southwestern USA Ponderosa Pine Forests
Source: PLoS One. 2015 Mar 13;10(3):e0118044. doi: 10.1371/journal.pone.0118044 (PMC4358824; doi:10.1371/journal.pone.0118044)

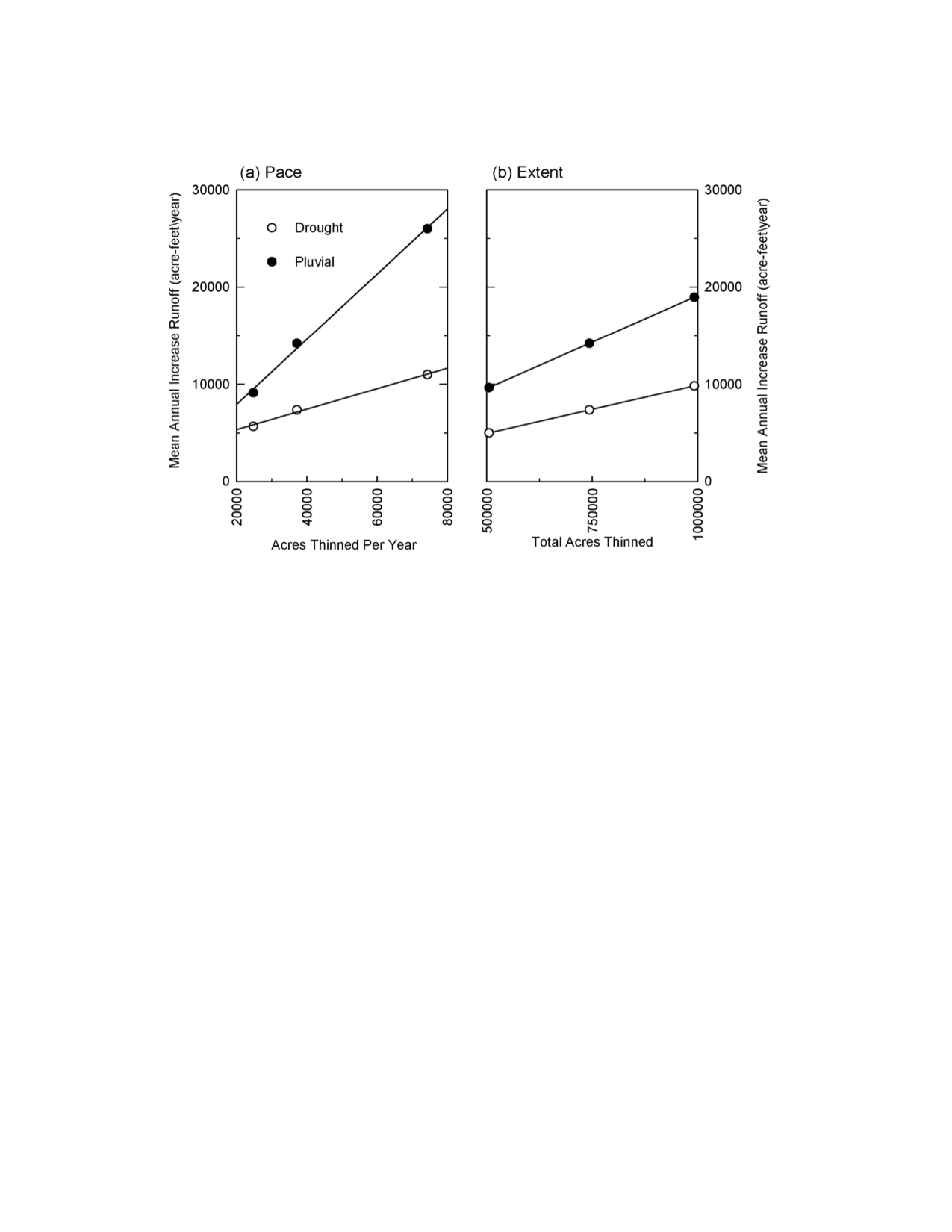

Supplement: S10 Fig — Effects of increasing (a) pace and (b) extent of thinning treatments of ponderosa pine forests in Salt-Verde watersheds on increases in mean annual runoff (acre-feet/year). In (a) total area thinned is held constant at 301,000 ha (743,000 acres) (scenarios: 35mid, 25mid, 15mid) to show influence of increasing the area thinned per year. In (b) duration of thinning treatments is held constant at 25 years (scenarios: 25low, 25mid, 25high) to show influence of increasing the total area thinned across the scenario. In order to illustrate scale effects, only increases in mean annual runoff are shown. Statistics describing annual variability in runoff in these scenarios is shown in Table 2 and illustrated graphically for 4FRI scenario in S8 Fig. (TIFF) [file pone.0118044.s001.tiff]
